# Luteolin for neurodegenerative diseases: a review

**DOI:** 10.1007/s43440-024-00610-8

**Published:** 2024-06-21

**Authors:** Dunuvilla Kavindi Jayawickreme, Cletus Ekwosi, Apurva Anand, Marta Andres-Mach, Piotr Wlaź, Katarzyna Socała

**Affiliations:** 1 Faculty of Biology and Biotechnology, Maria Curie-Skłodowska University, Akademicka 19, Lublin, 20–033 PL Poland; 2 Department of Experimental Pharmacology, Institute of Rural Health, Jaczewskiego 2, Lublin, 20-950 Poland; 3 Department of Animal Physiology and Pharmacology, Institute of Biological Sciences, Maria Curie-Skłodowska University, Akademicka 19, Lublin, 20–033 PL Poland

**Keywords:** Polyphenols, Apoptosis, Neuronal loss, Beta amyloid, Cognitive dysfunction, Learning and memory impairment, Parkinsonism

## Abstract

Neurodegenerative diseases such as Parkinson’s disease, Alzheimer’s disease, Huntington’s disease, and multiple sclerosis affect millions of people around the world. In addition to age, which is a key factor contributing to the development of all neurodegenerative diseases, genetic and environmental components are also important risk factors. Current methods of treating neurodegenerative diseases are mostly symptomatic and do not eliminate the cause of the disease. Many studies focus on searching for natural substances with neuroprotective properties that could be used as an adjuvant therapy in the inhibition of the neurodegeneration process. These compounds include flavonoids, such as luteolin, showing significant anti-inflammatory, antioxidant, and neuroprotective activity. Increasing evidence suggests that luteolin may confer protection against neurodegeneration. In this review, we summarize the scientific reports from preclinical in vitro and in vivo studies regarding the beneficial effects of luteolin in neurodegenerative diseases. Luteolin was studied most extensively in various models of Alzheimer’s disease but there are also several reports showing its neuroprotective effects in models of Parkinson’s disease. Though very limited, studies on possible protective effects of luteolin against Huntington’s disease and multiple sclerosis are also discussed here. Overall, although preclinical studies show the potential benefits of luteolin in neurodegenerative disorders, clinical evidence on its therapeutic efficacy is still deficient.

**Graphical abstract:**

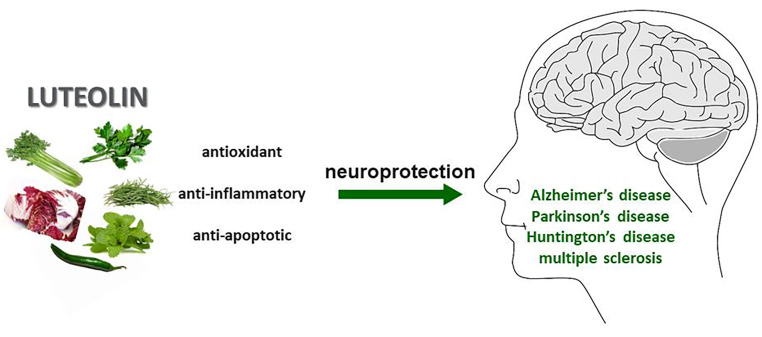

## Introduction

Neurodegenerative diseases are a heterogeneous group of chronic, usually age-related disorders that are characterized by the progressive loss of neurons, deformation of the neuron structure, or loss of neuron function [1]. They remain a major focus of scientific and clinical interest due to their increasing medical and social importance. Majority of the neurodegenerative diseases are characterized by the aggregation of intracellular proteins or their extracellular depositions (plaques) such as α-synuclein in Parkinson’s disease (PD), amyloid beta (Aβ)/tau aggregates in Alzheimer’s disease (AD), superoxide dismutase 1 (SOD 1) in amyotrophic lateral sclerosis (ALS), or huntingtin protein with tandem glutamine repeats in Huntington’s disease (HD) [2]. However, the etiology and exact pathogenesis of these devastating diseases is largely unknown.

The cause of most neurodegenerative diseases is considered as a combination of genetic and environmental factors. Both early and late-onset AD are dominated by genetic mutations on chromosome 1, 14, 21, and chromosome 19q13, respectively [3]. Similarly in PD, autosomal dominant genetic mutations such as leucine-rich repeat kinase 2 (LRRK2) and autosomal recessive mutations in the parkin genes (PRKN and PINK1) are common [4, 5]. Mutations at the HD gene include multiple repeats of the CAG repeat sequence [6]. Environmental factors associated with neurodegeneration are diet, lifestyle, and chemical exposures such as metals mercury, cadmium, inorganic arsenic, and pesticides (e.g., deltamethrin, paraquat, dieldrin and rotenone) [3, 7], whereas environmental factors that have been shown to reduce the incidence of neurodegeneration are nicotine, caffeine, statins, post-menopausal hormonal treatment and non-steroidal anti-inflammatory drugs such as ibuprofen [3, 8].

Although neurodegeneration is tightly bound with the concept of protein aggregation, it is not the sole contributor. Oxidative stress, neuroinflammation, programmed cell death, and abnormalities in the ubiquitin-proteasomal and autophagosomal/lysosomal systems are some key factors that can lead to neuronal cell death [9]. Briefly, oxidative stress is described as a condition where the natural antioxidant machinery in the body cannot overcome the increasing generation of reactive oxygen species (ROS) such as hydroxyl, alkoxyl, peroxyl, and hydrogen peroxide anions. The generation of ROS can be the result of various events such as free radical generation in the substantia nigra, abnormal metabolism of dopamine, abnormalities in the respiratory chain of the mitochondria, and increased production of Fe^2+^ as a result of Fenton’s reaction. Additionally, nitrosative stress can take place due to the generation of peroxynitrite from radical nitric oxide (NO) in the absence of antioxidant systems. The low availability of antioxidant enzymes (low SOD, glutathione peroxidase, and catalase) can make a cell vulnerable to oxidative stress [10, 11]. Neuroinflammation is a natural defense mechanism against pathogens; however prolonged neuroinflammation can be detrimental as it leads to neuronal death. Microglia and astrocytes are the two main types of cells associated with neuroinflammation. They produce pro-inflammatory cytokines, such as tumor necrosis factor alpha (TNF-α), interleukin (IL)-1β, -16, and chemokines, including the C-C motif chemokine ligand 2 (CCL2) and IL-18 to initiate neuroinflammation. The triggers that lead to the loss of neuroprotective functions and gain of neurotoxic functions are not exactly known but may depend on a variety of factors such as the stage and severity of neurodegeneration [12]. Programmed cell death is another area that is closely monitored with respect to neurodegeneration. A defect in the mechanism that otherwise only eliminates damaged or old populations of cells, may start a cascade of events that leads to the death of healthy neuronal cell populations. A group of cell stressors leading to axonal degeneration such as the c-Jun N-terminal kinase (JNK) pathway is a key event [13, 14]. Finally, it has been shown that the activity of the ubiquitin-proteasome system and the autophagy-lysosomal pathway declines, two of the major catabolic pathways of eukaryotic cells, progressively declines with aging. As a consequence, cells become overloaded with deleterious proteins contributing to the development of age-related diseases such as AD or PD [15, 16].

Thus, neurodegenerative diseases are multifactorial conditions involving various pathological mechanisms, and accordingly, they require complex therapeutic approaches. Most of the currently available drugs work symptomatically, without affecting the underlying cause of the disease [1]. Moreover, long-term use of these medications frequently results in significant adverse effects and a decrease in therapeutic efficacy. The development of new treatment strategies for most progressive neurodegenerative diseases is however challenging due to their above-mentioned multi-factorial pathogenesis and diverse disease course [17]. Plants have been valuable source of medicines for centuries and bioactive phytochemicals continue to represent a very attractive research field [18].

Luteolin (3′,4′,5,7-tetrahydroxyflavone; C_15_H_10_O_6_) belongs to a family of naturally occurring secondary metabolites called flavonoids characterized by a diphenylpropane structure (C6-C3-C6) [19], more specifically to the sub-group of flavonoids categorized as flavones. Structurally, it is a 15 C flavone with 2 benzene rings and one oxygen-containing ring arranged in the typical flavonoid structure mentioned above. As a tetrahydroxyflavone, luteolin has two hydroxyl groups on each of the two benzene rings [20, 21]. It was first isolated from a flowering plant called, *Reseda odorata* L. as a traditional medicinal potion in parts of Asia [22]. It is commonly found in vegetables from the Apiaceae family such as carrots (37.5 mg/kg dry weight), dried parsley (19.75 mg/100 g), and other vegetables including broccoli (74.5 mg/kg), green chilly (33.0 mg/kg), spinach (1.11 mg/100 g), and cabbage (0.4–0.6 mg/100 g). Herbs from the family of Lamiaceae, e.g., thyme (51 mg/100 g), fresh peppermint (11.33 mg/100 g), and others such as *Perilla frutescem* can be great sources of luteolin as well [21, 23]. Moreover, chrysanthemum flowers are consumed in the form of herbal teas and processed foods in East Asia as a source of antioxidants. The source of this antioxidant activity stems from the presence of luteolin in the flowers (1 mg of luteolin/100 mg of flowers) [24].

Luteolin occurs in the form of either aglycone or glucosides but glycosylated forms are more common. Its bioavailability was reported to be between 4.1% and 26% [20]. In the intestines, luteolin aglycone is absorbed into enterocytes by diffusion as it is lipophilic, whereas luteolin glycosides are converted first into luteolin aglycones. In the enterocytes and/or hepatocytes, luteolin is extensively metabolized into luteolin glucuronides and/or luteolin sulfates [20, 25]. Luteolin-3′-O-β-d-glucuronide and luteolin-3′-O-sulfate were identified as the most abundant metabolites in rat and human plasma, respectively [26]. After oral intake in rats, luteolin and its metabolites have been mostly distributed in the gastrointestinal tract, liver, kidneys, and lungs and biliary excretion was the main elimination pathway of the conjugated luteolin [25]. It has been demonstrated that luteolin given intraperitoneally (*ip*) to mice can readily cross the blood-brain barrier (BBB) and enter the brain [27]. To increase the bioavailability of luteolin, several delivery methods have been developed; the most thoroughly studied include lipid carriers like liposomes and nanoformulations [21].

**Fig. 1:**
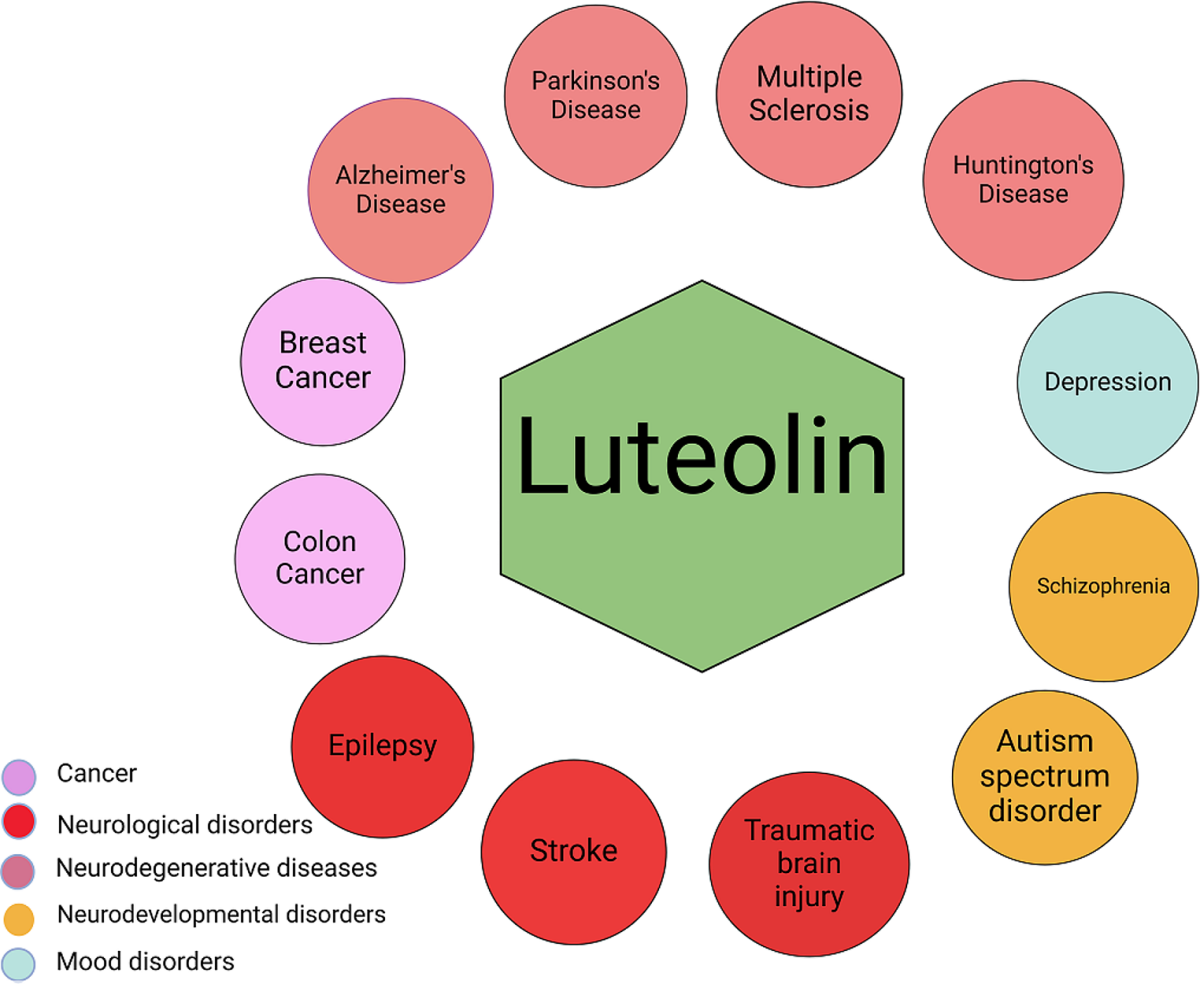
Potential therapeutic benefits of luteolin

Numerous studies show that luteolin possesses various health-beneficial properties including anti-apoptotic, anti-oxidant, anti-inflammatory, and neuroprotective effects. Consequently, it has gained considerable interest as a potential therapeutic agent in many disorders such as cancer and central nervous system (CNS) disorders including neurodegenerative diseases, traumatic brain injury, stroke epilepsy, schizophrenia, autism, and depression (Fig. 1) [11, 20, 21, 28–32]. In this review, we aimed to summarize the current knowledge on the potential beneficial effects of luteolin in four neurodegenerative diseases. We review and discuss data from both in vitro and in vivo preclinical experiments highlighting the potential mechanisms by which luteolin may confer neuroprotection in the aforementioned diseases.

## Mechanisms of protective action of luteolin in neurodegenerative diseases

Luteolin can exhibit beneficial effects in neurodegenerative disorders through various mechanisms including suppression of inflammatory processes, oxidative stress, and apoptosis. Moreover, some evidence shows that luteolin may reduce formation of Aβ plaques and enhance neuronal growth [33].

Neuroinflammation is one of the major hallmarks of neurodegeneration; therefore therapeutics with anti-inflammatory effects are beneficial in the treatment of these diseases. Scientists have studied the structure of luteolin to find evidence for its anti-inflammatory activities [34]. As a flavonoid, luteolin’s strength lies in the number of hydroxyl groups attached to the A and B rings. To be more precise, in the 5′ and 7′ positions of the A ring and 3′ and 4′ of the B ring. Many studies have been conducted to investigate the anti-inflammatory effects of luteolin [35–40]. Luteolin mediates anti-inflammatory responses by suppressing microglia and astrocytes and their downstream targets such as pattern recognition receptors; toll-like receptors 2 and 4 (TLR2 and TLR4) [41]. Luteolin has exerted its anti-inflammatory effect by inhibiting pro-inflammatory mediators such as cyclooxygenase-2 (COX-2), nitric oxide (NO), TNF-α, IL-β, IL-6, IL-8, IL-31, and IL-33 in several in vitro models of AD [42], PD [43, 44], MS [45], and in vivo models of AD [27, 46]. The inflammatory response produced by our immune systems is a well-coordinated mechanism including several pathways. Among them, the nuclear factor kappa-light-chain-enhancer of activated B cells (NF-κB), mitogen-activated protein kinase/activator protein-1 (MAPK/AP-1), and the Janus kinase signal transducer and activator of transcription (JAK-STAT) pathways have been identified as the targets of luteolin [40, 46–52]. The inhibition of the NF-кB pathway leads to the inhibition of a downstream target– β-site amyloid precursor protein cleaving enzyme (BACE1), which is a key mediator in forming Aβ fibrils in AD pathology [48, 53].

Luteolin exerts an anti-oxidant activity mainly by reducing ROS levels and increasing SOD activity in in vitro models of AD [54–56]. Luteolin can also increase the expression of antioxidant enzymes such as heme oxygenase-1 (HO-1) via the nuclear factor erythroid 2–related factor 2/ antioxidant responsive element (Nrf-2/ARE) complex activation, as it was observed in in vitro models of PD [44, 57] and H_2_O_2_-induced toxicity in in vitro models [58–60].

The anti-apoptotic effects of luteolin can be mediated *via* several mechanisms such as reducing the levels of caspase-3 and − 9 and improving the B-cell lymphoma protein 2/Bcl-2-associated X protein (Bcl-2/Bax) ratio, as it was reported in in vitro models of AD [61] and PD [62], as well as in vivo models of AD [63, 64] and PD [65, 66]. Among other flavones, luteolin exhibits the strongest cytoprotective activity by reducing the level of caspase-3 and PARP-1 activation and enhancing the unfolded protein response (UPR) pathway, leading to an increase in endoplasmic reticulum (ER) chaperone GRP78 and a decrease in the expression of UPR-targeted pro-apoptotic genes via the MAPK pathway. Additionally, a significant reduction in the p53 transcription factors such as p21, PUMA, and GADD45α were observed [62, 67, 68].

Luteolin also exerts neuroprotective effects by preventing Aβ-induced cell death via an anti-oxidant mechanism and upregulation of ER/ERK/MAPK signaling pathway in in vitro models of AD [55, 61, 69, 70]. There is also evidence that suggests that luteolin can directly influence the formation of Aβ plaques by selectively inhibiting the activity of N-acetyl-α-galactosaminyltransferase (ppGalNAc-T) isoforms [71] and in in vivo models of AD by selectively binding to Aβ fibrils, inactivating the glycogen synthase kinase-3 alpha (GSK-3α) isoform, suppressing Aβ and promoting tau disaggregation [27, 48, 63, 72, 73].

Additionally, luteolin has also a positive effect on neuronal growth and maintenance. Lin et al. [74] found that luteolin dose-dependently promoted the growth and differentiation of neurons both in terms of number and length and the up-regulation of growth-associated protein-43(GAP-43 expression) via the ERK-dependent pathway in PC12 cells. In a similar study, luteolin promoted phosphorylation and activation of cAMP response element-binding protein (CREB) leading to the increased miR-132 expression, and eventually neurite outgrowth in PC12 cells via MAPK/ERK- and cAMP-dependent protein kinase A (PKA) pathways [75].

**Fig. 2:**
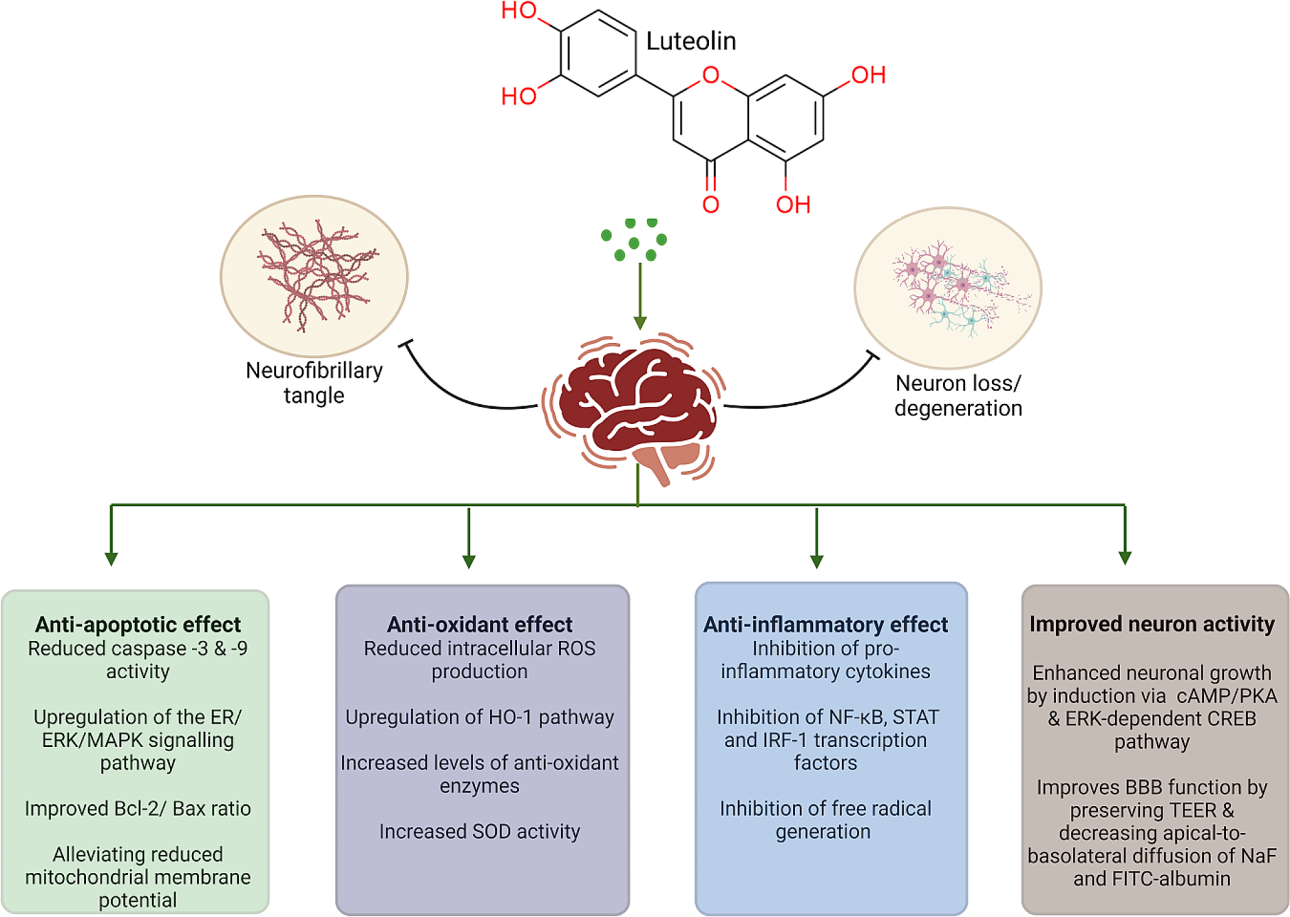
Schematic overview of the possible mechanisms underlying the neuroprotective effects of luteolin Abbreviations: BBB: blood-brain barrier; Bax: Bcl-2-associated X protein; Bcl-2: B-cell lymphoma protein 2; cAMP: cyclic adenosine monophosphate; ER: endoplasmic reticulum; ERK: extracellular signal-regulated kinase; ERK-dependent CREB: extracellular signal-regulated kinase-dependent cAMP response element-binding protein; FITC; fluorescein isothiocyanate; IRF-1: interferon regulatory factor 1; MAPK: mitogen-activated protein kinase; NaF: sodium fluoride; NF-κB: nuclear factor kappa-light-chain-enhancer of activated B cells; PKA; protein kinase A; ROS: reactive oxygen species; SOD: superoxide dismutase; STAT: signal transducer and activator of transcription; TEER: transepithelial electrical resistance

**Fig. 3:**
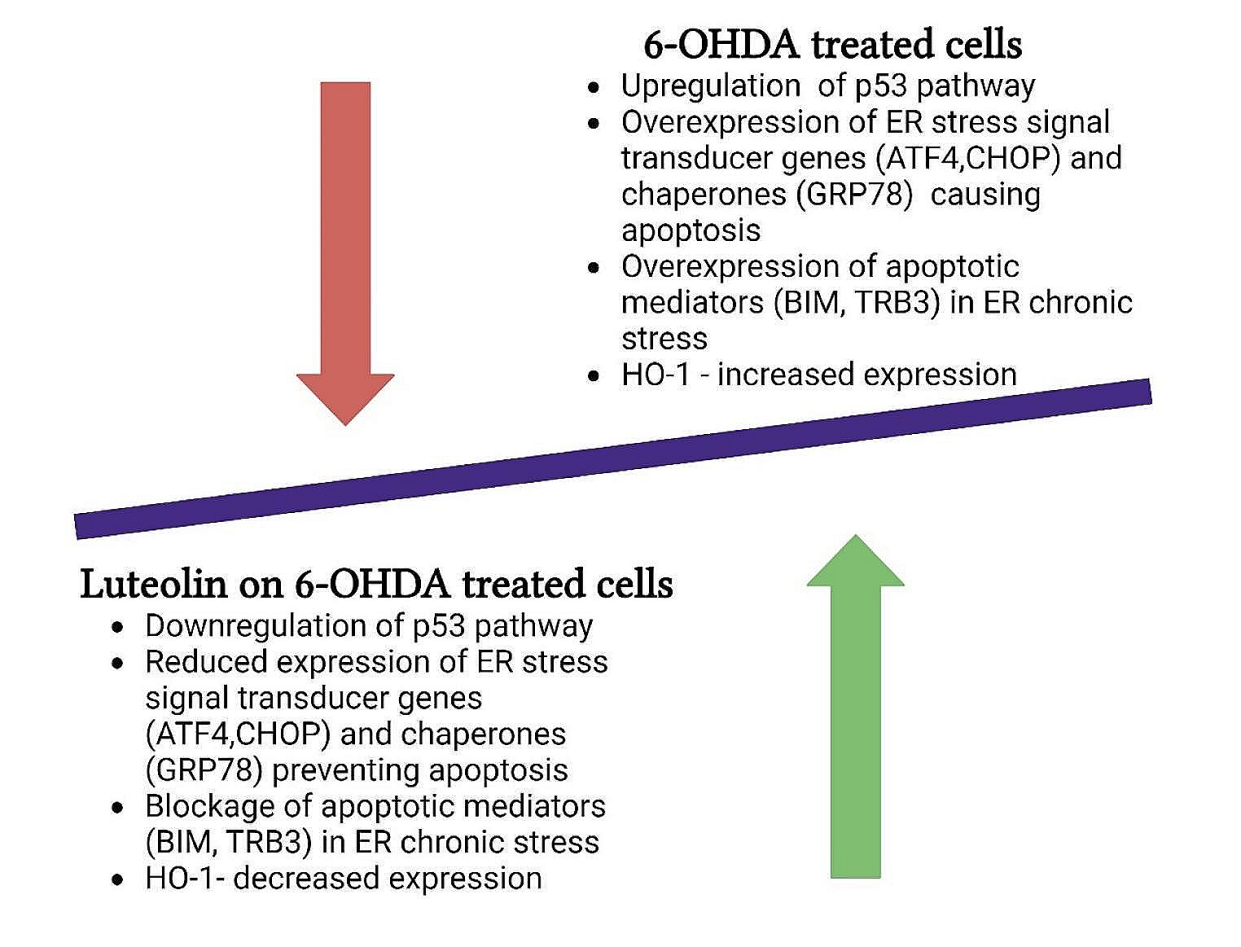
A summary of the effect of luteolin on the prevention of apoptosis in the 6-OHDA-indcued model of PD in vitro Abbreviations: ATF4: activating transcription factor 4; BIM: Bcl-2-like protein 11; CHOP: C/EBP homologous protein; ER: endoplasmic reticulum; GRP78: glucose-regulated protein 78; HO-1: heme oxygenase-1; p53: tumor protein p53; TRB3: tribbles homolog 3

More details on the possible mechanisms underlying the protective effects of luteolin in various in vitro and in vivo models of neurodegenerative diseases are provided in the next sections and summarized in Figs. 2 and 3.

## Preclinical studies on luteolin in neurodegenerative diseases

### Luteolin and Alzheimer’s disease

AD is the most common type of neurodegenerative disease and is responsible for the majority of dementia cases (60–80%) [1, 76]. The disease is progressive and it is marked by a gradual decline in memory, along with cognitive and executive dysfunction [76]. The pathology identified is Aβ peptide’s accumulation in the medial temporal lobe and neocortical structures of the brain resulting in neuritic plaques and neurofibrillary tangles [77]. It is estimated that more than 47 million individuals suffer from dementia globally, and the figure is expected to increase to 150 million by 2050 [78, 79]. AD can be classified into two types based on the age of onset. The first type is early-onset AD (EOAD), which is a rare form of the disease that accounts for around 1–6% of cases. EOAD typically affects people between the age group of 30–65 and is usually familial [77]. EOAD mutations consist of three primary autosomal dominant mutations; found on 3 different loci, the amyloid precursor protein (APP) gene on chromosome 21 which encodes for the amyloid precursor protein from which Aβ is derived, PSEN1 on chromosome 14 and PSEN2 gene on chromosome 1, the latter two genes encode the presenilin 1 and 2 subunits of the γ-secretase enzyme which catalyzes the cleavage of APP [80]. The second type is late-onset AD (LOAD), which is more common and typically affects people above the age of 65 [77]. The causes of LOAD are still unknown, but several factors have been shown to modify the risk of developing LOAD, such as environmental, lifestyle, ethnic, socioeconomic, and genetic factors. Both EOAD and LOAD can occur in families with a positive history of AD [79].

The two primary pathological features of end-stage AD are Aβ plaques and neurofibrillary tangles (NFTs). Amyloid plaques consist of aggregated misfolded Aβ protein that assembles from monomers into oligomers and fibrillar species which accumulate in the extracellular space [81]. In contrast, NFTs are made up of hyperphosphorylated tau protein and are formed within neuronal cell bodies and axons which can directly spread to other parts of the brain. These pathological changes begin to occur many years before onset of cognitive impairment and lead to significant neuronal loss in the hippocampal area and neocortex. NFTs are directly linked to progressive neuronal death, whereas Aβ species’ presence and accumulation are associated with other critical aspects of AD pathology as well, for example, soluble Aβ oligomers can cause mitochondrial and ER dysfunction, resulting in the overproduction of ROS, oxidative stress, and neuronal damage. Aβ oligomers and fibrils can also hinder functional synaptic connections and activity, which is believed to be a significant factor in cognitive decline, especially in the prodromal and early stages of AD [79]. The prevailing hypothesis is that the accumulation of Aβ peptide triggers a cascade of events that leads to tau hyperphosphorylation, tangle formation, neuronal dysfunction, and finally symptom development [82]. The amyloid cascade hypothesis which is based on the molecular defects observed in autosomal-dominant EOAD has been severely criticized due to the unsolved paradox: ‘Why do Aβ aggregate into fibrils?’, but it is clear that the Aβ sequence, Aβ concentration, and conditions that destabilize Aβ are important factors in aggregation [83, 84].

As discussed before, AD is a multifactorial condition and therefore numerous factors (both hereditary and lifestyle) apart from Aβ accumulation itself can contribute to or pose a risk to the development of this neurodegenerative disease [85, 86]. As the more common form is LOAD (99%), most research goes into combating the non-hereditary causes of AD. Oxidative stress, glucose metabolism, lipid metabolism, and inflammation are the main non-hereditary factors leading to the development of AD. Among all the mentioned, inflammation seems to have a heavy implication on the pathogenesis and progression of AD [86] and luteolin with its potent anti-inflammatory properties may produce beneficial effects in AD.

Prominent inflammation in AD has been observed through increased levels of proinflammatory cytokines, specifically TNF-α and IL-6, in both serum and brain tissue of AD patients compared to controls. Several studies have demonstrated the presence of immune cells and related proteins near Aβ plaques [87]. Latest clinical studies have reported higher expression of markers of astrogliosis and microgliosis, as well as of both peripheral and central neuroinflammation, in young AD cases as compared with old ones, indicating that all these phenomena are prominent at the earliest AD stage, and can even decrease with aging. These astonishing results have prompted scientists to rethink and approach different therapeutic targets [88].

Possible neuroprotective effects of luteolin have been studied in various preclinical models of AD. Firstly, a few in vitro studies investigated the effect of luteolin on the Aβ-induced toxicity. For example, luteolin was found to inhibit Aβ_25–35_-induced cell death and oxidative stress in rat cerebral microvascular endothelial cells and [89] and mouse cortical cells [69]. The protective effects of luteolin against Aβ_25−35_-induced toxicity have also been thoroughly investigated and confirmed by Wang et al. [61], who demonstrated that luteolin inhibited Aβ_25−35_-induced apoptosis of rat pheochromocytoma (PC12) cells by significantly downregulating the expression of Bax and caspase-3 and upregulating the expression of Bcl-2. Additionally, luteolin significantly upregulated the expression of estrogen receptor beta (Erβ) and phosphorylated extracellular signal-regulated protein kinases 1 and 2 (p-ERK1/2). These results indicate that luteolin activated the ER/ERK/MAPK signaling pathway to protect PC12 cells against Aβ_25−35_-induced cell apoptosis by selectively acting on Erβ [61].

Several investigations also tested the effect of luteolin on cell lines transfected with the ‘Swedish mutation of amyloid precursor protein’ (APPsw). Rezai-Zadeh et al. [73] carried out such an investigation to test the effect of luteolin on Aβ peptide generation in human ‘Swedish’ mutant APP transgene-bearing neuron-like cells (SweAPP) and Tg2576 mouse-derived neuronal cells. The results showed a strong inhibition of both Aβ peptides by luteolin, which was associated with a downregulation of γ-secretase cleavage activity. To establish the mechanism of action, the proteins involved with γ-secretase were studied and the obtained results showed that luteolin increased the level of serine 21 phosphorylated GSK-3α isoform as well as increased presenilin-1 carboxyl-terminal fragment phosphorylation in a time-dependent manner. These two events consequently disrupt the enzyme-substrate association with APP resulting in an anti-amyloidogenic effect. The experiment with the Tg2576 mouse model further confirmed the anti-amyloidogenic effect of luteolin (reduction of both soluble Aβ_1–40, 42_ isoforms) [73].

Luteolin also exhibited anti-oxidant, anti-apoptotic, and anti-amyloidogenic activities in the copper-induced neurotoxicity model in the human neuroblastoma SH-SY5Y cells carrying the APPsw [56]. In the study by Dragicevic et al. [54], the effects of 25 natural compounds (including luteolin) on amyloid-induced mitochondrial dysfunction in murine neuroblastoma N2a cells expressing the Swedish mutation were evaluated. Three separate assays were performed to determine overall mitochondrial function: ROS production, mitochondrial membrane potential (MMP), and ATP levels were assessed. Luteolin was on top of the list as it almost completely restored the mitochondrial function in the N2a-APPsw neuroblastoma cells (ROS production was decreased by 40%, MMP levels were restored close to control N2a levels (202%), and ATP levels were improved by 444%).

An interesting study was performed by Zhang et al. [42], who investigated the protective effects of luteolin on the BBB using human brain microvascular endothelial cells and human astrocytes under fibrillary Aβ_1–40_ (fAβ_1–40_)-damaged conditions. Luteolin improved BBB functioning and reduced the fAβ_1−40_-induced release of cytokines and inflammatory mediators. The anti-inflammatory activity was mediated through the p38 MAPK/NF-κB pathway [42].

It is also worth to mention about beneficial effect of luteolin on the inhibition of zinc-induced tau phosphorylation in human neuroblastoma SH-SY5Y cells. The mechanism of action was associated with the recovery of total phosphatase activity [70].

Furthermore, it has been shown that O-GalNAc glycosylation (initiated by the members of ppGalNAc-T family of APP is associated with the production of Aβ in AD [90]. Liu et al. [71] studied the effect of luteolin on the inhibition of ppGalNAc-T in several cell lines was studied. Luteolin showed a concentration-dependent inhibitory effect on the GalNAz signal in O-GalNAc glycosylation deficient (CHO-IdID) cells, indicating a potent inhibitory effect on O-GalNAc glycosylation. It also showcased specific inhibitory effects against O-GalNAc in HEK 293T and Jurkat cells. The evaluation of the inhibitory effects of luteolin on APP protein showed reduced ratio of the high-molecular-weight band of APP (APP-H; O-GalNAc-glycosylated APP) with a 75 and 80% reduction in HEK 293T cells and A549 cells, respectively. The mechanism of action was coupled with the inhibition of specific ppGalNAc-T isoforms in cells. Luteolin also inhibited Aβ production in Swedish-mutation APP stable cell line (HEK 293TAPP Swe cell). Finally, possible correlation between the inhibition of ppGalNAc-T and Aβ production by luteolin was investigated. In CHO-IdID cells, luteolin was able to reduce the level of restoration of Aβ, Aβ_40_, and Aβ_42_ by approximately 30% when treated with Gal and GalNAc, confirming the hypothesis about the correlation between the inhibition of ppGalNAc-T and Aβ production [71].

Not only in vitro but also in vivo studies provide evidence that luteolin may produce beneficial effects in AD. Animal studies showed that luteolin may improve cognitive decline which is a key symptom of AD. For instance, prolonged treatment with luteolin was shown to attenuate the cognitive impairment induced by chronic cerebral hypoperfusion (CCH) in rats [48, 91, 92]. Xu et al. [92] found that CCH caused dramatic inhibition of LTP formation in the hippocampus and luteolin was able to prevent the LTP impairment. It not only facilitated the phosphorylation of CREB in the hippocampus of naïve rats but also rescued the CCH-induced impairment of CREB activation. In the study by Fu et al. [48], attenuated cognitive dysfunction in CCH rats treated with luteolin was accompanied by reduced oxidative stress and neuroinflammation. Luteolin also down-regulated the expression of NF-κB and BACE1, as well as diminished the deposition of Aβ in the cortex and hippocampus. In addition, He and Chen [91] showed that luteolin ameliorated cognitive impairment in CCH rats through the modulation of the PI3K/Akt pathway.

The protective effect of luteolin against cognitive dysfunction was also reported in the streptozotocin- [93] and Aβ_25–35_-induced models of AD [94, 95]. In the Aβ_25–35_-induced model of AD in mice, luteolin attenuated cognitive impairment by, at least in part, modulating the microvascular function. Luteolin increased regional cerebral blood flow values, alleviated the leakage of the lumen of vessels, and protected the integrity of BBB [95]. Luteolin could also ameliorate cognitive deficits by regulating the cholinergic system activity, inhibiting oxidative stress [94, 95], and increasing the level of brain-derived neurotrophic factor (BDNF) and tyrosine kinase receptor (TrkB) expression in the cerebral cortex [95].

Furthermore, several reports showed neuroprotective effects of luteolin in genetic models of AD. Sawmiller et al. [27] studied the effects of luteolin on AD-like pathologies induced by traumatic brain injury in Tg2576 mice overexpressing the human APP gene carrying the Swedish mutation. Within 3 days, the untreated injured mice had significant increases in Aβ deposition, phosphorylated tau accumulation, GSK-3 activation, as well as elevated TNF-α and IL-1β levels, which is consistent with AD pathology. The luteolin-treated mice exhibited none of the AD-associated pathologies or elevations in the inflammatory cytokines [27]. The Tg2576 mouse model of AD was also used by Rezai-Zadeh et al. [73] who found that luteolin reduced GSK-3 activation and cerebral Aβ levels. In contrast to the study by Sawmiller et al. [27], luteolin supplementation for 29 weeks failed to improve cognitive impairment in APP23 mice that also overexpress human APP with the Swedish mutation. It did, however, alleviate the depression-like behavior and inhibited microglial activation by regulating the ER stress [96].

The neuroprotective effects of luteolin were also studied in a triple-transgenic mouse model of AD (3 × Tg-AD). This model is different from the previous ones as it exhibits both Aβ and tau pathology, as well as synaptic dysfunction [97]. In the study by Kou et al. [41], luteolin treatment dose-dependently improved spatial learning and alleviated memory deficits in 3 × Tg-AD mice. Luteolin also reduced histopathological changes and the number of dense Aβ plaques, inhibited over-activation of astrocytes, decreased expression of ER stress-related proteins, and reduced neuroinflammation. Specifically, it restored the increased levels of inflammatory markers and reduced the expression of p-NF-κB and phospho-p38 in the brain of AD mice. Likewise, He et al. [64] showed that luteolin supplementation significantly ameliorated memory and cognitive deficits in 3 × Tg-AD mice. This effect was accompanied by reduced generation and accumulation of Aβ, inhibited neuronal apoptosis, decreased oxidative stress, and attenuated mitochondrial dysfunction *via* peroxisome proliferator-activated receptor gamma (PPARγ) activation.

A similar positive effect of luteolin was observed in the transgenic Drosophila model of AD where the transgenic flies fed on different concentrations of luteolin showed a dose-dependent delay in the loss of climbing ability, increased lifespan, reduced oxidative stress, acetylcholinesterase activity and Aβ_42_ peptides accumulation [63].

Palmitoylethanolamide (PEA) is an endogenous molecule that acts as an anti-inflammatory mediator on several cells such as astroglia, microglia, oligodendroglia, and mast cells to maintain cellular homeostasis in the CNS and peripheral nervous system. Facchinetti et al. [88] conducted a study to demonstrate the effects of ultra-micronized PEA combined with luteolin (co-ultra PEALut; 10:1 by mass) in the Aβ_1–42_-induced model of AD in rats. The study was able to showcase the neuroprotective mechanisms of co-ultra PEALut by observing a decrease in GFAP levels (a biomarker of astrocytes) and a reduced increase in CD11b gene expression (a marker for activated microglial cells) in the hippocampus of the Aβ_1–42_-inoculated rats. Moreover, co-ultra PEALut prevented the Aβ_1–42_-induced increase in mRNA expression of inflammatory biomarkers as well as Aβ_1–42_-induced reduction of BDNF and GDNF mRNA levels [88].

The combination of luteolin with another compound– l-theanine (an amino acid found in tea) also improved AD-like symptoms in the Aβ_25–35_-treated rats. Luteolin in combination with l-theanine significantly improved memory function by potentiating the insulin signaling in the hippocampus and by reducing inflammation. Luteolin potentiated insulin signaling mainly via the pAkt/pGSK/pTau pathway, whereas l-theanine primarily reduced TNF-α [98].

Various formulations and drug delivery systems have been introduced to overcome limitations associated with poor solubility in water, low oral bioavailability, and extensive first-pass metabolism of luteolin. For example, luteolin-loaded bile-salt-based nano-vesicles (bilosomes) and luteolin-loaded chitosan decorated nanoparticles delivered intranasally have been developed as a strategy to enhance luteolin solubility and BBB permeability. Both luteolin bilosomes [99] and luteolin chitosomes [72] were found to ameliorate cognitive impairment in the streptozotocin-induced model of AD in mice. They also reduced oxidative stress and neuroinflammation, decreased Aβ aggregation and hyperphosphorylated-tau levels in the hippocampus, and increased neuronal survival [72, 99].

In summary, AD is the most common neurodegenerative disease in the world and its pathogenesis is tightly coupled with neuroinflammation and is estimated to rise in future shortly. This puts a big weight on the shoulders of scientists in the field of neurodegeneration to look for potential candidates to combat AD. As one of the key outcomes of luteolin is anti-inflammation, it consequently makes luteolin a worthy candidate. Upon in vitro and in vivo experimentation (summarized in Tables 1 and 2, respectively), it is evident that luteolin was able to inhibit microglial-associated inflammation pathways such as NF-κB, MAPK/AP-1 which produce pro-inflammatory cytokines which are associated with activating β-secretase and γ-secretase leading to stimulating Aβ formation. It was also observed that luteolin can restore cognitive impairment. Unfortunately, although preclinical data show clear-cut neuroprotective effects of luteolin in AD models, there are no human intervention studies or clinical trials investigating its potential beneficial effects in AD patients. Thus, clinical trials investigating its possible beneficial effects in AD patients are highly warranted.

**Table 1:** Summary of in vitro studies on neuroprotective effects of luteolin

Cell line	Treatment	Model	Main findings	Reference
*Alzheimer’s disease*				
Rat cerebral microvascular endothelial cells (CMECs)	0.1,1.0, and 10.0 μmol/L of luteolin co-treated with 100 μmol/L^1^ of Aβ_25–35_	Aβ_25–35_ induced model of AD	(1) Protective effects against Aβ_25–35_ toxicity by improving cell viability, reducing ROS production, and increasing SOD activity, (2) improving tight junctions of CMECS by maintaining TEER activity and regulating TXA2 and PGI2 secretion	[89]
Human neuroblastoma SH-SY5Y cells	0, 1.0 and 10 μM of luteolin co-treated with 300 μM of copper	β-Amyloid precursor protein Swedish mutation (APPsw) induced model of AD	(1) Anti- amyloidogenic effect by blocking the increased expression of AβPP and Aβ_1–42_ peptide secretion by copper, (2) anti-oxidant capacity by reducing intracellular ROS generation and increasing SOD activity; (3) anti- apoptotic effect by alleviating reduced levels of mitochondrial membrane potential and reduced caspase − 3 and − 9 activity	[56]
Human neuroblastoma SH-SY5Y cells	5–100 μM luteolin co-treated with 300 μM zinc	Zinc induced tau phosphorylation at Ser262/356	Attenuates zinc-induced tau hyperphosphorylation through alleviation of total phosphatase activity	[70]
Mouse cortical cells	3–30 μM of luteolin co-treated with 20 μM of Aβ_25–35_	Aβ_25–35_ induced model of AD	Protective effects against Aβ_25−35_ induced toxicity by inhibiting neuronal cell death	[69]
HEK 293T-APP Swedish mutant cells	0, 10, 20 and 30 μM of luteolin	–	(1) Reduction in the production of APP-H and the amounts of total Aβ, Aβ_40_, and Aβ_42_ in a dose-dependent manner, (2) reduction in sAPPα and sAPPβ levels	[71]
Human brain microvascular endothelial cells (hBMECs)	3.0, 10, 30 μM of luteolin co-treated with 20 μM of fAβ_1–40_	In vitro BBB model of AD (fAβ_1–40_)	(1) Improves BBB function by preserving TEER and decreasing apical-to-basolateral diffusion of NaF and FITC-albumin, (2) anti-inflammatory effect by inhibiting TNF-α, IL-1β, IL-6, IL-8, and the expression of COX-2 through p38 MAPK/NF-κB pathway	[42]
Human astrocytes (hAs)	3.0, 10, 30 μM of luteolin co-treated with 20 μM of fAβ_1–40_	In vitro BBB model of AD (fAβ_1–40_)	(1) Improves BBB function by preserving TEER and decreasing apical-to-basolateral diffusion of NaF and FITC-albumin, (2) anti-inflammatory effect by inhibiting TNF-α, IL-1β, IL-6 and IL-8 and the expression of COX-2 through p38 MAPK/NF-κB pathway	[42]
Rat pheochromocytoma cells (PC12)	10^− 6^, 10^− 5^, 10^− 4^, 10^− 3^, 10^− 2^, 10^− 1^, 1 and 10 μmol/L of luteolin co-treated with 20 μmol/L of Aβ_25–35_	Aβ_25–35_ induced model of AD	Protective effects against Aβ_25–35_ induced toxicity by inhibiting cell apoptosis through the upregulation of ER/ERK/MAPK signaling pathway	[61]
Murine neuroblastoma cells (N2a)	1 μM of luteolin	Swedish mutation (APPsw) induced model of AD	Restoration of mitochondrial dysfunction by reducing ROS levels and increasing mitochondrial membrane potential and ATP production	[54]
	5, 10, 20 and 40 μM of luteolin	Swedish mutation (APPsw) induced model of AD	Anti-amyloidogenic effect by the downregulation of γ-secretase via inhibition of GSK-3 and phosphorylation of PS1 CTF proteins	[73]
*Parkinson’s disease*				
Rat pheochromocytoma cells (PC12)	5 μM luteolin and co-treated with 100 μM MPP^+^ (a concentration causing 50% cell death)	MPP^+^-induced model of PD	Anti-oxidant capacity by Nrf2-activation through MEK-ERK 1/2 signaling pathway	[57]
	3.1, 12.5, and 50 μM luteolin 250 μM 6-OHDA	6-OHDA-induced model of PD	(1) Inhibition of the 6-OHDA induced apoptosis by increasing the Bcl-2 (anti-apoptotic protein) and decreasing the Bax (pro-apoptotic protein) ratio, (2) inhibition of the p53 transcription factor.	[67]
	10 and 20 μM luteolin with 100 mM 6-OHDA	6-OHDA-induced model of PD	(1) Inhibition of apoptosis by reducing the level of caspase-3, by reducing ER stress related gene ATF4, blocking UPR pathway (CHOP and GRP78) (2) antioxidative effects by reducing ROS levels	[62]
Rat primary microglia	1–50 μM luteolin and co-treated with 20 mM of rotenone	Rotenone-induced neurodegeneration	Antioxidant and anti-inflammatory effects by inhibiting IL-1b, LDH release and Lrrk2 gene expression without affecting TNF-a and Pink1 levels, increasing the redox modulating effectors (such as Nrf-2 and Trx1) and cytoprotective mediators (Park2) at transcriptional levels.	[44]
Human neuroblastoma cells (SH-SY5Y)	10–200 μM luteolin-7-O-glucoside with 2 mM MPP^+^	MPP^+^-induced model of PD	(1) Inhibition of apoptosis by improving Bcl-2/Bax ratio and inhibiting cleaved caspase-3, (2) prevented suppression of ERK1/2 and STAT3	[65]
	2.5–80 μM luteolin with 100 mM MPP^+^	MPP^+^-induced model of PD	(1) Inhibition of apoptosis by decreased expression of pro-apoptotic proteins (such as Bax, cleaved caspase-3, and cytochrome c) and increased expression of anti-apoptotic protein Bcl-2, (2) decreased expression of synaptic proteins GAP43 and synapsin-1, (3) Suppressed Cdk5 Activity, (4) inhibited MPP^+^-induced reduction in phosphorylated Erk1/2, Drp1, Fak, Akt, and GSK3β	[104]
	5 μM luteolin with 6-OHDA	6-OHDA-induced model of PD	(1) Increased expression of HRD1 and its stabilizer SEL1L at both mRNA and protein levels, (2) significantly reduced 6-OHDA-induced cell death, apoptosis (measured by cleaved caspase-3 levels), and ER stress (measured by CHOP mRNA levels), (3) neuroprotective effect by modulating the HRD1 and SEL1L pathways	[105]
Rat glioblastoma cells (C6)	5 μM luteolin and co-treated with 100 μM MPP^+^	MPP^+^-induced model of PD	Anti-oxidant capacity by Nrf2-activation through MEK-ERK 1/2 signaling pathway	[57]
*Huntington disease*				
Mutant (STHdhQ111/Q111, clone 109–1 A) striatal cells	5 and 10 μM UA or 10 and 20 μM of luteolin or luteolin derivatives (Lut-C1, Lut-C4, Lut-C6 and Lut-C10)	HD mouse striatal cells	(1) Decreased ROS levels in HD striatal cells after luteolin and luteolin derivatives treatment, (2) activated the Nrf2 pathway by Lut-C4 and Lut-C6 in HD striatal cells, (3) Lut-C6 promoted mRNA expression of antioxidant-related enzymes in HD cells	[109]
*Multiple sclerosis*				
Peripheral blood mononuclear cells (PBMCs) isolated from MS patients	0, 0.2, 1, 5,10, 25, 50 μM of luteolin, 2 IU of IFN-β, or a combination of 0. 0.2, 1, 5, 10 μM of luteolin, and 2 IU of IFN-β	Peripheral blood mononuclear cells	(1) Luteolin reduced, in a dose-dependent manner, the proliferation of PBMCs, (2) modulated the levels of IL-1β and TNF-α released by PBMCs, (3) reduced the MMP-9/TIMP-1 ratio (4) in combination with IFN-β, had additive effects in modulating cell proliferation	[45]
Primary culture of oligodendrocyte precursors	PEALut (1–10 μM)	Oligodendrocyte precursor cells	PEALut promoted morphological development of OPCs and total protein content without affecting proliferation	[114]
Oligodendrocyte Progenitor Cell Culture	PEALut (10 μM)	Oligodendrocyte precursor cells	PEALut promotes oligodendrocyte differentiation by the increase of MBP and CNPase and Tyro3 mRNAs	[115]
*Other studies*				
Rat pheochromocytoma cells (PC12	5, 10, and 20 μM luteolin	–	(1) Anti-oxidant capacity by the up-regulation of the HO-1 and Nrf2-ARE pathway, (2) enhanced neuronal growth through the ERK pathway	[74]
	20 μM luteolin	–	Enhanced neuronal growth by induction of immature and mature forms of miR-132 via up-regulation of cAMP/PKA and ERK-dependent CREB pathway	[75]
	10, 25 and 50 μg/ml of luteolin with 400 μM H_2_O_2_	H_2_O_2_-induced apoptosis	(1) Inhibition of apoptosis by increased expression of Bcl-2 and decreased expression of Bax, (2) antioxidant effects by enhanced PI3K/Akt pathway in response to H_2_O_2_	[59]
	5–20 μM luteolin with 0.1–40 μM arsenite	Arsenite-induced apoptosis	(1) Antioxidant effects by reducing ROS, (2) enhanced caspase-3 activity and γ-H2AX expression, (3) inhibition of α-synuclein by siRNA	[129]
BV-2 microglial cell	0–50 μmol/L luteolin with LPS for 4–24 h	LPS-induced neuroinflammation	Anti-inflammatory effect by reducing the level of pro-inflammatory mediators of microglia such as TNF-α, nitrite, and PGE2	[130]
	0–50 μM luteolin with 10 ng/ml or 50 ng/ml LPS	LPS-induced neuroinflammation	(1) Anti-inflammatory and anti-apoptotic effect by reducing the level of pro-inflammatory mediators such as IL-6, Cxcl 10, IrF7, Ifi44 of microglia through the AP-1, IRF1, STAT (independent of NFkB) pathways and reducing pro-apoptotic markers such as CHOP, Gadd153, (2) potent modulator of the transcriptome of resting/LPS-activated microglia; (3) reduced NO secretion	[131]
Rat primary microglia	10, 15, and 20 μM luteolin and co-treated with 100 ng/ml LPS or (10 U/ml) IFN-γ	LPS-induced neuroinflammation	(1) Anti-inflammatory effect by inhibiting NO, TNF-α and IL-1β production, (2) inhibition of NF-κB, STAT and IRF-1 transcription factors, (3) inhibition of free radical generation and activation of phosphatase activity	[37]
	5, 10 and 20 μM luteolin and co-treated with 200 μM H_2_O_2_	H_2_O_2_-induced cytotoxicity	Antioxidant effect by increasing levels of anti-oxidant enzymes (catalase and glutathione) consequently reducing ROS levels	[60]
Primary cultured rat cortical cells	0.3–30 μM luteolin and co-treated with 100 μM H_2_O_2_ or xanthine (0.5 mM)/xanthine oxidase (10 mU/mL)	H_2_O_2_- or xanthine/xanthine oxidase-induced oxidative damage	(1) Antioxidant effect by attenuation of ROS generation, HO-1 upregulation through p38 MAPK, JNK and Akt signaling pathways (2) inhibition of apoptosis by Ser112 phosphorylation of Bad (anti-apoptotic protein) and inhibition of the cleavage of pro-caspase 3	[58]
Human hepatoma (HepG2)	1.56–6.25μM luteolin	–	Antioxidant effects through upregulation of Nrf2 and Nrf2-regulated genes involved in HO-1 expression; inhibition of NO production, reduction of iNOS and Cpla2 proteins	[132]
Mast cells from mouse fetal liver cells (MC/9)	Exposed to oxygen and glucose deprivation and co-treated with 1 nm–100 μM of PEALut	Brain ischemia model	Anti-inflammatory effect by inhibiting TNF-α and IL-1β levels production through the inhibition of NF-κB pathway	[133]

Abbreviations: 6-OHDA: 6-hydroxydopamine; Aβ: amyloid beta; Aβ1–42: amyloid beta 1–42; Aβ25–35: amyloid beta 25–35; Aβ40: Amyloid beta 40; Aβ42: Amyloid beta 42; AβPP: amyloid beta precursor protein; Akt: protein kinase B; AP-1: activator protein-1; APP-H: high-molecular weight band of APP; APPsw: Swedish mutation of amyloid precursor protein; ATF4: activating transcription factor 4; Bax: Bcl-2-associated X protein; Bcl-2: B-cell lymphoma 2; BV-2: brain macrophage 2; Cdk5: cyclin-dependent kinase 5; CHOP: C/EBP homologous protein; CMECs: cerebral microvascular endothelial cells; COX-2: cyclooxygenase-2; CREB: cAMP response element-binding protein; Cxcl 10: C-X-C motif chemokine ligand 10; Drp1: dynamin-related protein 1.; ER: endoplasmic reticulum; ERK: extracellular signal-regulated kinase; ERK1/2: extracellular signal-regulated kinase 1/2; fAβ1–40: fibrillar amyloid beta 1–40; Fak: focal adhesion kinase; FITC: fluorescein isothiocyanate; Gadd153: also known as chop (mentioned earlier); GAP43: growth-associated protein 43; GSK-3: glycogen synthase kinase-3; GSK3β: glycogen synthase kinase-3 beta; GRP78: glucose-regulated protein 78; HD: Huntington’s disease; HEK 293T-APP: human embryonic kidney 293T– amyloid precursor protein; HepG2: hepatocellular carcinoma derived from human liver; HO-1: heme oxygenase-1; HRD1: HMG-CoA reductase degradation protein 1; IFN-β: interferon-beta; IL-1β: interleukin-1 beta; IL-6: interleukin-6; IL-8: interleukin-8; iNOS: inducible nitric oxide synthase; IrF7: interferon regulatory factor 7; Ifi44: interferon-induced protein 44; JNK: c-Jun N-terminal kinase; LDH: lactate dehydrogenase; LPS: lipopolysaccharide; Lrrk2: leucine-rich repeat kinase 2; Lut-C1: luteolin derivatives bearing 3-alkyl chains of 1 carbon; Lut-C4: luteolin derivatives bearing 3-alkyl chains of 4 carbon; Lut-C6: luteolin derivatives bearing 3-alkyl chains of 6 carbon; Lut-C10: luteolin derivatives bearing 3-alkyl chains of 10 carbon; MAPK: mitogen-activated protein kinase; MBP: myelin basic protein; MEK-ERK: mitogen-activated protein kinase (MAPK) signaling pathway involving the proteins MEK (MAPK/ERK kinase) and ERK (extracellular signal-regulated kinase); miR-132: microRNA-132; MMP-9: matrix metalloproteinase-9; MPP^+^: 1-methyl-4-phenylpyridinium; N2a: neuro-2a; NFkB: nuclear factor kappa-light-chain-enhancer of activated B cells; NO: nitric oxide; Nrf2: nuclear factor erythroid 2–related factor 2; Nrf2-ARE: nuclear factor erythroid 2-related factor 2– antioxidant response element; OPCs: oligodendrocyte progenitor cells; PC12: pheochromocytoma 12; PBMCs: peripheral blood mononuclear cells; PEALut: palmitoylethanolamide-luteolin combination; PGE2: prostaglandin E2; PI3K/Akt: phosphoinositide 3-kinase/protein kinase B (Akt); Pink1: PTEN-induced putative kinase 1; PKA: protein kinase A; p38: p38 mitogen-activated protein kinase; PS1: presenilin-1 (PS1) carboxyl terminal fragment (CTF); ROS: reactive oxygen species; SEL1L: suppressor/enhancer of Lin-12-like; Ser112: amino acid serine at position 112; Ser262/356: amino acid serine at position 262/356 SH-SY5Y: human neuroblastoma; siRNA: small interfering RNA; SOD: superoxide dismutase; STHdhQ111/Q111: mouse model mimicking Huntington’s disease STAT: signal transducer and activator of transcription; STAT3: signal transducer and activator of transcription 3; TEER: transendothelial electrical resistance; TIMP-1: tissue inhibitor of metalloproteinase-1; TNF-α: tumor necrosis factor alpha; TRX1: thioredoxin 1; TRB3: tribbles homolog 3; TXA2: thromboxane A2 Tyro3: tyrosine-protein kinase 3 UPR: unfolded protein response; γ-H2AX: gamma-histone 2AX

**Table 2:** Summary of in vivo studies on luteolin in neurodegenerative disease models

Animals	Sex	Treatment	Model	Behavioral tests	Main findings	Reference
*Alzheimer’s disease*						
Sprague-Dawley rats	M	50, 150 and 450 mg/kg of luteolin (*po*) for 4 weeks after the surgery	Chronic cerebral hypoperfusion	Morris water maze test	(1) Attenuated cognitive deficits and reduced LTP impairment, (2) facilitated phosphorylation of CREB in hippocampus of normal rat, (3) and reduced chronic cerebral hypoperfusion-induced impairment of CREB activation	[92]
Sprague-Dawley rats	M	50, 100, and 200 mg/kg (*po*) of luteolin for 8 weeks after the surgery	Chronic cerebral hypoperfusion (CCH) model	Morris water maze test	(1) Ameliorated cognitive impairment, (2) reduced oxidative stress and inflammation, (3) decreased expression of BACE1 and NF-κB, and (4) reduced Aβ_(1–40)_ and Aβ_(1–42)_ levels	[48]
Sprague-Dawley rats	M	50 and 100 mg/kg of luteolin (*po*) for 3 months after the surgery	Chronic cerebral hypoperfusion	Morris water maze test, object recognition test	(1) Attenuated cognitive deficits, (2) improved PI3Kp110α and PI3Kp85 expression and stimulated phosphorylation of Akt	[91]
Wistar rats	M	10 and 20 mg/kg of luteolin for 5 days	Streptozotocin-induced model of AD	Morris water maze test	(1) Ameliorated memory impairment, (2) iprevented reduction of the thickness of CA1 pyramidal layer	[93]
Kunming mice	M	5 and 10 mg/kg (*po*) of luteolin for 8 days	Aβ_(25–35)_-induced model of AD	Morris water maze test	(1) Attenuated cognitive deficits, (2) alleviated leakage of the lumen of vessels and protected BBB integrity, (3) decreased AChE level and activity, (4) increased rCBF values, (5) increased expression of occludin, ZO-1, and claudin-5, (6) reduced ROS level, and (7) increased BNDF level and TrkB expression in the cortex	[94]
Sprague-Dawley rats	NS	100 and 200 mg/kg (*po*) of luteolin for 17 days	Aβ_1–40_-induced model of AD	Morris water maze test, passive avoidance test	(1) Attenuated cognitive deficits, (2) decreased AChE activity (3) increased ACh level, (4) increased ChAT activity, (5) reduced oxidative stress, (6) increased Bcl-2/Bax ratio in the hippocampus.	[95]
C57BL/6 N mice	M	80 mg/kg of luteolin for 2 weeks	Aβ_1–40_-induced model of AD	–	(1) Attenuated neuronal apoptosis, (2) reduces accumulation of β-amyloid and synaptic dysfunctions, (3) diminished expression of activated p-NF-kB and other inflammatory markers, (4) regulation of expression of p-JNK/P-38 and expression of specific markers of activated microglia and astrocytes	[46]
Tg2576 mice	NS	20 mg/kg (*ip*) of luteolin for 15 days before TBI induction	TBI in the amyloid depositing Tg2576 mouse model of AD	–	Reduced Aβ deposition, glycogen synthase-3 activation, phospho-tau accumulation, and pro-inflammatory cytokines level.	[27]
Tg2576	F/M	20 mg/kg of luteolin (*ip*) for 30 days	Amyloid depositing Tg2576 mouse model of AD	–	(1) Reduced GSK-3 activation, (2) reduction of cerebral Aβ levels	[73]
APP23 mice	F/M	20 mg/kg of luteolin (*ip*) for 29 weeks	Genetic mouse model of AD	Fear conditioning test, Y maze test, tail suspension test, forced swim test	(1) Improved depressive-like behavior, (2) lack of improvement of memory impairment, (3) inhibited microglial activation through regulation of ER stress	[96]
3 × Tg-AD mice	F/M	20 and 40 mg/kg (*ip*) of luteolin for 3 weeks	Genetic mouse model of AD	Morris water maze test	(1) Ameliorated memory impairment, (2) inhibited astrocyte overactivation, (3) reduced neuroinflammation, (4) decreased ER stress markers GRP78 and IRE1α	[41]
3 × Tg-AD mice		20 and 40 mg/kg (*ip*) of luteolin for 8 weeks	Genetic mouse model of AD	Novel object recognition task, step-down avoidance test, and open field test	(1) Ameliorated memory and cognitive impairment, (2) inhibited Aβ generation, (3) reduced mitochondrial damage, (4) reduced neuronal apoptosis	[64]
Drosophila	F/M	5, 10, 15 and 20 μM of luteolin for 30 days	transgenic Drosophila model of AD	Climbing assay, activity pattern assay	(1) A dose-dependent delay in the loss of climbing ability and activity, (2) increase in a life span (3) reduction of Aβ42 expression, (4) reduced oxidative stress, (5) reduced AChE, caspase 3 and 9 activity	[63]
Sprague-Dawley rats	M	5 mg/kg (*ip*) of co-ultra PEALut (10:1 by mass) for 14 days	Aβ_(1–42)_-induced model of AD	–	(1) Prevented astrogliosis and microgliosis, (2) reduced expression of proinflammatory genes, and (3) increased GDNF and BDNF mRNA levels.	[88]
Sprague-Dawley rats	M	50 mg/kg of luteolin or 100 mg of l-theanine or 25 mg/kg of luteolin + 50 mg/kg of l-theanine in a high-fat diet for 8 weeks	Aβ_25–35_-induced model of AD	Morris water maze test, passive avoidance test	(1) Luteolin + l-theanine improved memory function better than either alone, (2) potentiated hippocampal insulin signaling and reduced inflammation, (3) luteolin potentiated insulin signaling via the pAkt/pGSK/pTau pathway	[98]
Albino Swiss mice	M	50 mg/kg (*in*) of luteolin or luteolin bilosomes (equivalent to 50 mg/kg, *in*) for 21 days	Streptozotocin-induced model of AD	Y-maze test and Morris water maze test	(1) Ameliorated cognitive impairment, (2) decreased levels of Aβ aggregation and hyperphosphorylated-tau, (3) increased neuronal survival, (4) reduced oxidative stress and neuroinflammation	[99]
Albino Swiss mice	M	2 mg/kg (*in*) of luteolin or luteolin chitosomes (equivalent to 2 mg/kg of luteolin, *in*) for 21 days	Streptozotocin-induced model of AD	Y-maze test and Morris water maze test	(1) Ameliorated cognitive impairment, (2) decreased levels of Aβ aggregation and hyperphosphorylated-tau, (3) increased neuronal survival, (4) reduced oxidative stress and neuroinflammation	[72]
*Parkinson’s disease*						
C57BL/6 mice	M	30 mg/kg (*po*) of luteolin-7-O-glucosid for 15 days	MPTP-induced model of PD	Pole test, traction test	(1) Improved PD symptoms (bradykinesia, muscle strength, and balancing capacity), (2) prevented depletion of TH positive neurons in substantia nigra and neurofibers in striatum, (3) attenuated gliosis in substantia nigra	[65]
*Huntington’s disease*						
Drosophila	F/M	25, 50, 75 and 100 μM of luteolin for 33 days	transgenic Drosophila model of HD	Climbing assay	(1) A dose-dependent delay in the loss of climbing ability, (2) a dose dependent significant decrease in the oxidative stress (3) a positive interaction between mHTT and luteolin from molecular docking studies	[110]

Abbreviations: Aβ: amyloid beta; ACh: acetylcholine; AChE; acetylcholinesterase; AD: Alzheimer’s disease; BACE1: β-site APP cleaving enzyme 1; Bax: Bcl-2-associated X protein; BBB: blood-brain barrier; Bcl2: B-cell lymphoma protein 2; BDNF: brain-derived neurotrophic factor; ChAT: choline acetyltransferase; co-ultra PEALut: co-ultramicronized palmitoylethanolamide and luteolin; CREB: AMP response element-binding protein; ER: endoplasmic reticulum; F: female; GDNF: glial cell line-derived neurotrophic factor; GRP78; glucose-regulated protein 78; GSK3: glycogen synthase kinase 3; HD: Huntington’s disease; IRE1: inositol enzyme 1; *in*: intranasally; *ip*: intraperitoneally; JNK: c-Jun N-terminal kinase; LTP: long-term potentiation; M: male; mHTT: mutant huntingtin protein; MPTP: 1-methyl-4-phenyl-1: 2: 3,6-tetrahydropyridine; NF-κB: Nuclear factor kappa-light-chain-enhancer of activated B cells; NS: not specified; pAkt; phosphorylated protein kinase B; PD: Parkinson’s disease; pGSK: phosphorylated glycogen synthase kinase 3; PI3K: phosphatidylinositol 3-kinase; *po*: per os; rCBF: regional cerebral blood flow; ROS: reactive oxygen species; TBI: traumatic brain injury; TH: tyrosine hydroxylase; TrkB: tyrosine kinase receptor; ZO-1: zonula occludens-1

### Luteolin and Parkinson’s disease

PD is the second most common neurodegenerative disease. It is a complex, progressive disorder dominated by movement disabilities such as tremors and bradykinesia but also includes cognitive abnormalities such as PD dementia, depression, and other non-motor symptoms related to the autonomic nervous system. Statistically, in Europe, prevalence and incidence rates for PD are estimated at approximately 108–257/100 000 and 11–19/100 000 per year, respectively [5, 100]. The etiology of PD is complex as there is both the aspect of genetic and environmental factors that have been shown to contribute to the disease [101]. The most frequently occurring autosomal dominant gene mutation is the G2019S missense mutation in LRRK2. Autosomal recessive mutations associated with PD include mutations in the parkin genes such as PRKN and PINK1. Missense mutations in these genes lead to mitophagy. There are also examples of sporadic PD such as microtubule associated protein Tau (MAPT mutations) and glucocerebrosidase loss of function mutations [4, 5]. Pesticides such as rotenone and paraquat and other environmental toxins have been linked with causing PD by inducing oxidative stress.

α-Synuclein is a key protein involved in PD pathology. It is a 140 amino acid-long monomer found in the cytosol. In the presence of lipid droplets or lipid bilayers, it undergoes a conformational change by binding to the lipid components to give a folded α-helical secondary structure that is capable of forming dimers and oligomers. Post-translational modifications such as phosphorylation, ubiquitination and nitration have been associated with α-synuclein aggregation. Together with the lipid association and post-translational modifications, α-synuclein aggregates to form the Lewy bodies [102].

Luteolin has been shown to counteract key aspects of PD pathogenesis including apoptosis and neuronal death, oxidative stress, mitochondrial dysfunction [11, 103], and neuroinflammation that is directly linked to the non-motor symptoms of PD. The anti-inflammatory properties of luteolin have been thoroughly studied in in vitro and in vivo models, as reviewed elsewhere [50, 52]. Here, we focus on experimental data from in vitro and in vivo PD models.

Firstly, the protective effects of luteolin were studied in in vitro 1-methyl-4-phenylpyridinium (MPP^+^)-induced model of PD. Luteolin attenuated the MPP^+^-induced apoptosis in PC12 cells [57], rat glioblastoma cells [57], and human SH-SY5Y cells [104]. The protection against MPP⁺-induced neurotoxicity was associated with activation of Nrf2 pathway (a crucial defense mechanism against oxidative stress), via the ERK1/2 signaling pathway [57], inhibition of the mitochondrial ROS-dependent oxidative stress, and enhancement of the Erk1/2/Drp1 and Fak/Akt/GSK3β signaling pathways [104]. In addition, Qin et al. [65] conducted a study on the effect of luteolin-7-O-glucoside (LUT-7G) on the MPP^+^-treated SH-SY5Y cells. According to the obtained data, LUT-7G protected SH-SY5Y cells against MPP^+^-induced apoptosis by regulating apoptosis-related protein expression. Specifically, LUT-7G treatment decreased nuclear condensation, elevated the MPP^+^-induced decline of Bcl-2/Bax ratio, and suppressed the MPP^+^-induced increase of cleaved caspases 3, which are both markers of apoptosis.

Several in vitro studies also investigated the neuroprotective effects of luteolin in the 6-hydroxydopamine (6-OHDA) model of PD (Fig. 3). Guo et al. [67] demonstrated the impact of luteolin on the reduction of nuclear condensation and fragmentation decreasing PC12 cell apoptosis induced by 6-OHDA. In the case of neuronal cell apoptosis, luteolin significantly reduced the expression of the pro-apoptotic (Bax) gene, while increasing the anti-apoptotic (Bcl) gene. It also significantly suppressed the enhanced Bax/Bcl-2 ratio and down-regulated the enhanced mRNA expression of p53 induced by 6-OHDA. Hu et al. [62] conducted a similar investigation on the effect of luteolin on a 6-OHDA model of PD in PC12 cells, in which luteolin reduced the negative effects of 6-OHDA exposure, including ROS overproduction, cytotoxicity, and caspase-3 activation leading to apoptosis. Additionally, luteolin reduced cell cycle arrest and transcription of p53 target genes, as well as downregulated the unfolded protein response. A recent study revealed that luteolin significantly reduced the 6-OHDA-induced cell death, apoptosis, and ER stress in SH-SY5Y cells by targeting ER stress responses [105].

While numerous in vitro experiments demonstrated that luteolin may be effective against PD, to date only two studies have been conducted to evaluate the beneficial effects of luteolin in animal models of PD. Siddique et al. [66] investigated the effect of luteolin on different aspects associated with PD in the transgenic *Drosophila melanogaster* expressing human α-synuclein. They found that a 24-day treatment with luteolin dose-dependently delayed the loss of climbing ability and improved the activity pattern in transgenic fly. The behavioral effects were accompanied by reduced oxidative stress markers and caspase-3 and 9 activities in the brains of PD flies. Importantly, luteolin was also able to increase the dopamine level by increasing the activity of tyrosine hydroxylase (TH), which is a key enzyme responsible for generating dopamine [66]. In another study, a protective effect of LUT-7G in the 1-methyl-4-phenyl-1, 2, 3,6-tetrahydropyridine (MPTP)-induced model of PD in mice was studied. A 15-day treatment with LUT-7G attenuated the MPTP-evoked bradykinesia, and improved muscle strength, and balancing capacity. LUT-7G also protected dopaminergic neurons from MPTP-induced toxicity. It increased the number of TH-positive cells in the substantia nigra and the density of TH positive neurofibers in the striatum. In addition, a significant suppression of gliosis in substantia nigra was observed, which suggests the anti-neuroinflammatory effects of LUT-7G in the MPTP-induced model of PD [65].

To sum up, PD is another good example of a disease that falls into the category of ‘cannot be cured’. This problem stems primarily due to the lack of sufficient knowledge of the pathogenesis of the disease. However, as we keep discovering the molecular mechanisms that underlie PD, we keep looking for effective treatment with a lack of adverse effects (a key issue with most available drug-based treatment). Luteolin has a big advantage over synthetic drugs as it is a naturally occurring flavonoid found in commonly consumed fruits and vegetables and has shown very few to no side effects. The range of neuroprotective roles luteolin plays is quite evident in in vitro models (Table 1). However, a lack of sufficient data still exists concerning the in vivo preclinical (Table 2) and clinical studies; therefore, the question of using luteolin as a drug against PD is still under experimentation.

### Luteolin and Huntington’s disease

Huntington’s disease (HD) is an autosomal dominant neurodegenerative disease manifesting by uncontrolled body movements and cognitive impairment. It is an inherited disorder that causes gradual breakdown of neurons in parts of the brain and may ultimately result in death within 15 to 20 years after diagnosis. The pathophysiology of HD is based on CAG triplet repeats in the huntingtin gene (HTT), leading to an expanded polyglutamine stretch in the huntingtin (Htt) protein [106]. Although there has been great progress in HD pathogenesis, currently available neuroprotective treatment strategies are still insufficient.

Neuroinflammation plays a key role in the development and progression of HD, and the disease may begin before significant neuronal loss occurs during the disease. It is closely linked with a wide range of biological impairments such as oxidative stress, which may induce mitochondrial dysfunction, and mitochondrial damages and lead to gliosis, with loss of astrocytes and oligodendrocytes, and in neuronal death and atrophy of brain tissues [107].

The type of HD treatment depends primarily on the patient’s clinical symptoms: (1) a progressive motor disorder; (2) progressive cognitive disorder culminating in dementia; and (3) psychiatric disorders including depression, anxiety, apathy, obsessive-compulsive behaviors, outbursts, addictions, and occasionally psychosis. Some of these symptoms can be treated non-pharmacologically, whereas the others require the use of medications [106] as well as add-on therapy with natural substances, e.g., flavonoids [108].

Only a few studies (Table 1) investigated the potential beneficial effects of luteolin in HD. The protective effect of luteolin and four luteolin derivatives was indicated by Oliveira et al. [109] in striatal cells derived from HD knock-in mice expressing mutant Htt *versus* wild-type striatal cells. Results they obtained showed a significant decrease of caspase-3-like activity and intracellular ROS and increased nuclear levels of phospho(Ser40)- Nrf2 and Nrf2/ARE transcription after treatment with 2 out 4 luteolin derivatives. Additionally, it was confirmed that one of luteolin derivatives enhanced SOD1 mRNA and SOD activity and glutamate-cysteine ligase catalytic subunit (GCLc) mRNA and protein levels, whereas another one induced mRNA levels of GCLc only in mutant striatal cells. The obtained results suggest that also luteolin derivatives may be important in the search for new antioxidant strategies in HD.

Molecular docking studies by Hasan Siddique et al. [110] have shown the effectiveness of luteolin as an anti-inflammatory agent. The results obtained for free energy of binding along with the non-covalent interactions proved a good interaction between luteolin and the mutant huntingtin protein (mHTT). Additionally, using a transgenic Drosophila model of HD they observed a dose-dependent delay in the loss of climbing ability and a decrease in the oxidative stress compared to the control flies after 33 days of luteolin supplementation [110] (Table 2).

Luteolin as an anti-oxidant plays a key role in the reduction and elimination of oxidative stress which is one of the major factors affecting the CNS (increased cell death, mitochondrial dysfunction, reduced neuronal plasticity and neurogenesis, increased autoimmune responses) in neurodegenerative diseases including HD [103, 111]. Taking into consideration the antioxidant properties of luteolin (protection from oxidative damage by scavenging free radicals and increasing the activity of antioxidant enzymes) it can be concluded that the use of this natural supplement in the treatment of HD may slow down or even stop the progression of the disease. Nevertheless, there are not enough scientific reports so far regarding the relationship between luteolin and mHTT and further advanced studies are needed to develop a new potent drug candidate able to reduce the aggregation of this pathogenic protein in the body.

### Luteolin and multiple sclerosis

Confirmed antioxidant, anti-inflammatory, and neuroprotective activities of luteolin certainly enabled research to assess the effectiveness of this substance in other neurodegenerative disorders multiple sclerosis (MS). MS is a chronic demyelinating disease of the CNS, in which progressive neuroinflammation induces lesions throughout the white and gray matter of the brain and spinal cord. It affects approximately 2.5 million people worldwide [112, 113]. The most important symptoms of MS are visual disorders (double vision, vestibular symptoms, dysphagia, dysarthria), and motor, cognitive, and mental disorders (depression, anxiety) [113].

First, in vitro investigations provided by Sternberg et al. [45] on peripheral blood mononuclear cells (PBMCs) isolated from MS patients showed that luteolin dose-dependently reduced PBMCs proliferation as well as production of IL-1β, TNF-α, and matrix metalloproteinase-9 (MMP-9), the inflammatory factors that are crucial in MS. Additionally, luteolin in combination with interferon-beta therapy, had and additive impact on cell proliferation, IL-1β, TNF-α, MMP-9 and tissue inhibitor of metalloproteinase-1.

Considering the problem of the remyelination of the myelin-producing oligodendrocytes in MS disease, Barbierato et al. [114] examined the ability of co-ultra PEALut to promote progression of oligodendrocyte progenitor cells (OPCs) into a more differentiated phenotype. The results they obtained showed that co-ultra PEALut promoted the morphological development of OPCs without affecting proliferation, which suggests that co-ultra PEALut may be a potential new drug candidate in the treatment of inflammatory demyelinating disorders.

Further, in vitro research on the OPC culture derived from rat pup cortex by Facci et al. [115] indicated an association between co-ultra PEALut-induced OPC maturation and tyrosine-protein kinase (Tyro3) receptor upregulation, which may be of great significance for the remyelination process.

While the above-mentioned findings (summarized in Table 1) are very promising, it is important to note that studies on the potential neuroprotective effects of luteolin in MS are at a very preliminary stage as yet.

### Human studies on luteolin in CNS disorders

Despite promising findings from preclinical in vitro and in vivo studies, the potential of luteolin in the treatment and/or prevention of neurodegenerative diseases has not been widely studied in clinical settings thus far.

Camptocormia, also known as the bent spine syndrome, when the trunk of a patient bends forward when walking or standing, is a common axial symptom of PD [116]. Camptocormia mainly arises in the later stages of PD. Its response to levodopa is very poor and there are no other effective treatment options [117]. Interestingly, a formulation which combined luteolin with PEA was found to significantly improve dyskinesia and reduce camptocormia in a 68-year PD patient. It was concluded that luteolin in this combination increases the stability of PEA and enhance the neuroprotective effects. Thus, co-ultra PEALut may be a promising adjuvant therapy for patients with PD to treat both dyskinesia and camptocormia but this needs confirmation in large-scale studies [116].

Frontotemporal dementia is a devastating neurodegenerative disorder manifested by progressive impairment in behavior, executive function, and language [118]. Recently, a randomized controlled trial on co-ultra PEALut in frontotemporal dementia has been completed. This study aimed to evaluate a global disease severity and various executive functions including cognition changes (ClinicalTrials.gov Identifier: NCT04489017). Unfortunately, the outcomes are not available as yet (as of May 2024).

It is noteworthy that there is also some initial clinical evidence on the beneficial effects of luteolin in other CNS conditions. Theoharides et al. [119] reported an uncontrolled open case series of 37 children aged 4–14 years (29 boys and 8 girls) suffering from autism spectrum disorder (ASD) who received a mixture of luteolin (100 mg) with the related flavonoids quercetin (70 mg) and rutin (30 mg). After 4 months of treatment, a significant improvement in various areas was reported. Specifically, 50% of the children had increased eye contact and attention to directions, 30–50% of children showed improvement in retaining learned tasks, 10% of the children started speaking words and even sentences, and lower incidence of hyperactivity and aggression. The same luteolin-containing dietary formulation was tested in a group of 50 children (4–10 years old; 42 boys and 8 girls) with ASD in a prospective open-label clinical trial. Positive changes in adaptive functioning and in the overall behavior were reported after a 26-week treatment [120]. Children that improved the most after dietary supplementation with this luteolin-containing formulation showed reduced IL-6 and TNF serum levels [121], which suggest that the beneficial effects were associated with anti-inflammatory action.

Brain fog is a clinical symptom where an individual has difficulty with memory, concentration, decision-making, and subjective cognitive impairment or behavioral changes. It was discovered as a persistent symptom by almost a third of COVID-19 patients. In a clinical study where De Luca et al. [122] investigated the effect of co-ultra PEALut on ‘brain fog’ in COVID-19 patients, some light was shed on the potential of luteolin on cognitive impairment (mainly memory loss) in humans. The study group was patients with a confirmed history of COVID-19 in the age group 40–50 years (43 women and 26 men). Three months after treatment with co-ultra PEALut a statistical reduction in brain fog was reported. The mechanism of action was deduced to be interconnected to neuroinflammation [122].

Co-ultra PEALut was also reported to improve the neurological status of human stroke patients. In an observational study, a cohort of 250 stroke patients (118 women and 132 men) undergoing neurorehabilitation were treated with co-ultra PEALut. After 30 days of supplementation, a significant improvement in neurological status, cognitive abilities, the degree of spasticity, independence in daily living, and pain was reported [123].

Although the above-mentioned data should be interpreted with caution due to their observational nature, they do show that luteolin-containing formulations are well-tolerated [119, 120, 123] and have the potential to attenuate cognitive deficits. This encourages further clinical studies, also in patients suffering from neurodegenerative disorders.

In January 2022, a double-blind, placebo-controlled, randomized clinical trial of the efficacy of luteolin (300 mg daily for 12 weeks) in patients with schizophrenia has been registered at clinicaltrials.gov (NCT05204407). The study will measure the effect of luteolin treatment on global psychopathology, cognitive impairments; antioxidant stress, levels of inflammatory markers, as well as positive and negative symptoms of schizophrenia. It is worth noticing here that luteolin has not been extensively studied in schizophrenia. Molecular docking and functional assays showed that luteolin has a potent antagonist effect on dopamine D_4_ receptors– the prime target for antipsychotic drugs [124]. In in vitro model of maternal immune activation (MIA), luteolin reduced astrogliosis and produced neuroprotective effects suggesting that it may attenuate the MIA-induced neurodevelopmental abnormalities occurring in ASD or schizophrenia [125]. Thus, the anti-inflammatory and neuroprotective action of luteolin may bring positive effects in schizophrenia that shares some features of neurodegenerative diseases [126].

## Future perspectives and conclusions

As reviewed above, accumulating evidence shows that luteolin may confer neuroprotection against various neurodegenerative disorders, especially in Alzheimer’s disease. Although the results of the current preclinical studies are very promising, much effort is needed to fully evaluate the therapeutic potential of luteolin. Such studies should include not only the assessment of the neuroprotective effects of luteolin across validated animal models of neurodegenerative disease but also the evaluation of its safety, tolerability, long-term adverse effects, pharmacokinetic profile, and interaction with CNS drugs. Moreover, the potential bidirectional interference of luteolin with the gut microbiota should be characterized since intestinal microbiomes can influence the bioavailability of orally taken polyphenols. Luteolin may also alter the gut microbiota composition, which consequently can affect its bioavailability and efficacy. In further perspective, well-designed clinical studies are warranted to verify the possible benefits of luteolin in neurodegenerative diseases.

Given the large increase in the aging population in recent decades, there is no doubt that the growing number of patients suffering from neurodegenerative diseases has become a major health problem. Apart from aging, there are many other causes of irreversible neuronal damage and death such as genetic mutations, pathologic proteins, and environmental factors [127]. From molecular point of view, the process of neurodegeneration is strongly related to neuroinflammation and oxidative stress [128].

Finding effective therapies that can delay the onset or progression of neurodegenerative diseases, especially AD and PD, is a primary goal and challenge for neuroscience research. Certainly, searching for and testing natural substances with neuroprotective, antioxidant, and anti-inflammatory properties may be an extremely important stage in the development of potential add-on therapies supporting the treatment of a given neurodegenerative disease. Flavones, including luteolin have shown therapeutic potential and can contribute to the treatment of neurodegenerative diseases. Current studies involving luteolin as a therapeutic agent are promising and strongly encourage further research on flavonoids showing neuroprotective activity.

## Data Availability

Data sharing does not apply to this article as no new data were generated or analyzed in this study.

## Abbreviations

6-OHDA: 6-hydroxydopamine
Aβ: Amyloid beta
AD: Alzheimer’s disease
ALS: Amyotrophic lateral sclerosis
AP-1: Activator protein-1
APP: Amyloid precursor protein
APPsw: Swedish mutation of amyloid precursor protein
ARE: Antioxidant responsive element
ASD: Autism spectrum disorder
BACE-1: Beta site amyloid precursor protein cleaving enzyme
Bax: Bcl-2-associated X protein
BBB: Blood-brain barrier
Bcl-2: B-cell lymphoma protein 2
BDNF: Brain-derived neurotrophic factor
CCH: Chronic cerebral hypoperfusion
CNS: Central nervous system
co-ultra PEALut: Ultra-micronized PEA combined with luteolin
COX-2: Cyclooxygenase-2
CREB: Camp response element-binding protein
EOAD: Early-onset Alzheimer’s disease
Erβ: Estrogen receptor beta
ER: Endoplasmic reticulum
ERK: Extracellular signal-regulated protein kinase
fAβ_1–40_: Fibrillary amyloid beta (1–40)
GCLc: Glutamate-cysteine ligase catalytic subunit
GSK-3α: Glycogen synthase kinase-3 alpha
HD: Huntington’s disease
HO-1: Heme oxygenase-1
HTT: Huntingtin
IL-1β: Interleukin-1 beta
IL-16: Interleukin-16
JAK-STAT: Janus kinase signal transducer and activator of transcription
LOAD: Late-onset Alzheimer’s disease
LRRK2: Leucine-rich repeat kinase 2
LUT-7G: Luteolin-7-O-glucoside
MAPK: Mitogen-activated protein kinase
mHTT: Mutant huntingtin protein
MIA: Maternal immune activation
MMP-9: Matrix metalloproteinase-9
MMP: Mitochondrial membrane potential
MPTP: 1-methyl-4-phenyl-1, 2, 3,6-tetrahydropyridine
NF-κB: Nuclear factor kappa-light-chain-enhancer of activated B cells
NFTs: Neurofibrillary tangles
Nrf2: Nuclear factor erythroid 2–related factor 2
OPCs: Oligodendrocyte progenitor cells
p-ERK1/2: Phosphorylated extracellular signal-regulated protein kinases 1 and 2
PBMCs: Peripheral blood mononuclear cells
PC12: Pheochromocytoma cell line 12
PD: Parkinson’s disease
PEA: Palmitoylethanolamide
ppGalNAc-T: Polypeptide N-acetyl-α-galactosaminyltransferase
ROS: Reactive oxygen species
SOD: Superoxide dismutase
TH: Tyrosine hydroxylase
TLR: Toll-like receptor
TNF-α: Tumor necrosis factor alpha
TrkB: Tyrosine kinase receptor

## References

[1] Lamptey RNL, Chaulagain B, Trivedi R, Gothwal A, Layek B, Singh J. A review of the common neurodegenerative disorders: current therapeutic approaches and the potential role of nanotherapeutics. Int J Mol Sci. 2022;23(3):1851. 35163773 10.3390/ijms23031851 PMC8837071

[2] Sweeney P, Park H, Baumann M, Dunlop J, Frydman J, Kopito R, et al. Protein misfolding in neurodegenerative diseases: implications and strategies. Transl Neurodegener. 2017;6:6. 28293421 10.1186/s40035-017-0077-5 PMC5348787

[3] Elbaz A, Dufouil C, Alpérovitch A. Interaction between genes and environment in neurodegenerative diseases. C R Biol. 2007;330:318–28. 17502288 10.1016/j.crvi.2007.02.018

[4] Elkouzi A, Vedam-Mai V, Eisinger RS, Okun MS. Emerging therapies in Parkinson disease– repurposed drugs and new approaches. Nat Rev Neurol. 2019;15:204–23. 30867588 10.1038/s41582-019-0155-7 PMC7758837

[5] Kouli A, Torsney KM, Kuan WL. Parkinson’s disease: etiology, neuropathology, and pathogenesis. In: Stoker TB, Greenland JC, editors. Parkinson’s Disease: Pathogenesis and clinical aspects. Codon Publications, Brisbane (AU); 2018. 30702842

[6] Myers RH. Huntington’s disease genetics. NeuroRx. 2004;1:255–62. 15717026 10.1602/neurorx.1.2.255 PMC534940

[7] Chin-Chan M, Navarro-Yepes J, Quintanilla-Vega B. Environmental pollutants as risk factors for neurodegenerative disorders: Alzheimer and Parkinson diseases. Front Cell Neurosci. 2015;9:124. 25914621 10.3389/fncel.2015.00124 PMC4392704

[8] Oertel W, Schulz JB. Current and experimental treatments of Parkinson disease: a guide for neuroscientists. J Neurochem. 2016;139(Suppl 1):325–37. 27577098 10.1111/jnc.13750

[9] Dugger BN, Dickson DW. Pathology of neurodegenerative diseases. Cold Spring Harb Perspect Biol. 2017;9. 10.1101/cshperspect.a028035 PMC5495060 28062563

[10] Knight JA. Reactive oxygen species and the neurodegenerative disorders. Ann Clin Lab Sci. 1997;27:11–25. 8997453

[11] Siddique YH. Role of luteolin in overcoming Parkinson’s disease. BioFactors. 2021;47:198–206. 33443305 10.1002/biof.1706

[12] Kwon HS, Koh SH. Neuroinflammation in neurodegenerative disorders: the roles of microglia and astrocytes. Transl Neurodegener. 2020;9:42. 33239064 10.1186/s40035-020-00221-2 PMC7689983

[13] Andreone BJ, Larhammar M, Lewcock JW. Cell death and neurodegeneration. Cold Spring Harb Perspect Biol. 2020;12. 10.1101/cshperspect.a036434 PMC6996453 31451511

[14] Moujalled D, Strasser A, Liddell JR. Molecular mechanisms of cell death in neurological diseases. Cell Death Differ. 2021;28:2029–44. 34099897 10.1038/s41418-021-00814-y PMC8257776

[15] Behl T, Kumar S, Althafar ZM, Sehgal A, Singh S, Sharma N, et al. Exploring the role of ubiquitin-proteasome system in Parkinson’s disease. Mol Neurobiol. 2022;59:4257–73. 35505049 10.1007/s12035-022-02851-1

[16] Bustamante HA, González AE, Cerda-Troncoso C, Shaughnessy R, Otth C, Soza A, et al. Interplay between the autophagy-lysosomal pathway and the ubiquitin-proteasome system: a target for therapeutic development in Alzheimer’s disease. Front Cell Neurosci. 2018;12:126. 29867359 10.3389/fncel.2018.00126 PMC5954036

[17] Wareham LK, Liddelow SA, Temple S, Benowitz LI, Di Polo A, Wellington C, et al. Solving neurodegeneration: common mechanisms and strategies for new treatments. Mol Neurodegener. 2022;17:23. 35313950 10.1186/s13024-022-00524-0 PMC8935795

[18] Srivastava P, Yadav RS. Efficacy of natural compounds in neurodegenerative disorders. Adv Neurobiol. 2016;12:107–23. 27651251 10.1007/978-3-319-28383-8_7

[19] Gendrisch F, Esser PR, Schempp CM, Wölfle U. Luteolin as a modulator of skin aging and inflammation. BioFactors. 2021;47:170–80. 33368702 10.1002/biof.1699

[20] Daily JW, Kang S, Park S. Protection against Alzheimer’s disease by luteolin: role of brain glucose regulation, anti-inflammatory activity, and the gut microbiota-liver-brain axis. BioFactors. 2021;47:218–31. 33347668 10.1002/biof.1703

[21] Taheri Y, Sharifi-Rad J, Antika G, Yılmaz YB, Tumer TB, Abuhamdah S, et al. Paving luteolin therapeutic potentialities and agro-food-pharma applications: emphasis on in vivo pharmacological effects and bioavailability traits. Oxid Med Cell Longev. 2021;2021:1987588. 34594472 10.1155/2021/1987588 PMC8478534

[22] Chagas M, Behrens MD, Moragas-Tellis CJ, Penedo GXM, Silva AR, Gonçalves-de-Albuquerque CF. Flavonols and flavones as potential anti-inflammatory, antioxidant, and antibacterial compounds. Oxid Med Cell Longev. 2022;2022:9966750. 36111166 10.1155/2022/9966750 PMC9470311

[23] Miean KH, Mohamed S. Flavonoid (myricetin, quercetin, kaempferol, luteolin, and apigenin) content of edible tropical plants. J Agric Food Chem. 2001;49:3106–12. 11410016 10.1021/jf000892m

[24] Takara T, Yamamoto K, Suzuki N, Yamashita SI, Lio SI, Kakinuma T, et al. Effects of luteolin-rich chrysanthemum flower extract on purine base absorption and blood uric acid in Japanese subjects. Funct Foods Health Dis. 2022;12:12. 10.31989/ffhd.v12i1.863

[25] Deng C, Gao C, Tian X, Chao B, Wang F, Zhang Y, et al. Pharmacokinetics, tissue distribution and excretion of luteolin and its major metabolites in rats: metabolites predominate in blood, tissues and are mainly excreted via bile. J Funct Foods. 2017;35:332–40. 10.1016/j.jff.2017.05.056

[26] Hayasaka N, Shimizu N, Komoda T, Mohri S, Tsushida T, Eitsuka T, et al. Absorption and metabolism of luteolin in rats and humans in relation to in vitro anti-inflammatory effects. J Agric Food Chem. 2018;66:11320–9. 30280574 10.1021/acs.jafc.8b03273

[27] Sawmiller D, Li S, Shahaduzzaman M, Smith AJ, Obregon D, Giunta B, et al. Luteolin reduces Alzheimer’s disease pathologies induced by traumatic brain injury. Int J Mol Sci. 2014;15:895–904. 24413756 10.3390/ijms15010895 PMC3907845

[28] Nabavi SF, Braidy N, Gortzi O, Sobarzo-Sanchez E, Daglia M, Skalicka-Woźniak K, et al. Luteolin as an anti-inflammatory and neuroprotective agent: a brief review. Brain Res Bull. 2015;119:1–11. 26361743 10.1016/j.brainresbull.2015.09.002

[29] Goyal A, Solanki K, Verma A, Luteolin. Nature’s promising warrior against Alzheimer’s and Parkinson’s disease. J Biochem Mol Toxicol. 2024;38:e23619. 10.1002/jbt.23619 38091364

[30] Savino R, Medoro A, Ali S, Scapagnini G, Maes M, Davinelli S. The emerging role of flavonoids in autism spectrum disorder: a systematic review. J Clin Med. 2023;12. 10.3390/jcm12103520 PMC10219283 37240625

[31] Sur B, Lee B. Luteolin reduces fear, anxiety, and depression in rats with post-traumatic stress disorder. Anim Cells Syst (Seoul). 2022;26:174–82. 36046028 10.1080/19768354.2022.2104925 PMC9423864

[32] Wu X, Xu H, Zeng N, Li H, Yao G, Liu K, et al. Luteolin alleviates depression-like behavior by modulating glycerophospholipid metabolism in the hippocampus and prefrontal cortex of LOD rats. CNS Neurosci Ther. 2024;30:e14455. 37715585 10.1111/cns.14455 PMC10916417

[33] Kempuraj D, Thangavel R, Kempuraj DD, Ahmed ME, Selvakumar GP, Raikwar SP, et al. Neuroprotective effects of flavone luteolin in neuroinflammation and neurotrauma. BioFactors. 2021;47:190–7. 33098588 10.1002/biof.1687

[34] Odontuya G, Hoult JR, Houghton PJ. Structure-activity relationship for antiinflammatory effect of luteolin and its derived glycosides. Phytother Res. 2005;19:782–6. 16220571 10.1002/ptr.1723

[35] Jang S, Kelley KW, Johnson RW. Luteolin reduces IL-6 production in microglia by inhibiting JNK phosphorylation and activation of AP-1. Proc Natl Acad Sci USA. 2008;105:7534–9. 18490655 10.1073/pnas.0802865105 PMC2396685

[36] Kang OH, Choi JG, Lee JH, Kwon DY. Luteolin isolated from the flowers of Lonicera japonica suppresses inflammatory mediator release by blocking NF-kappaB and MAPKs activation pathways in HMC-1 cells. Molecules. 2010;15:385–98. 20110898 10.3390/molecules15010385 PMC6257122

[37] Kao TK, Ou YC, Lin SY, Pan HC, Song PJ, Raung SL, et al. Luteolin inhibits cytokine expression in endotoxin/cytokine-stimulated microglia. J Nutr Biochem. 2011;22:612–24. 21036586 10.1016/j.jnutbio.2010.01.011

[38] Kim JS, Jobin C. The flavonoid luteolin prevents lipopolysaccharide-induced NF-kappaB signalling and gene expression by blocking IkappaB kinase activity in intestinal epithelial cells and bone-marrow derived dendritic cells. Immunology. 2005;115:375–87. 15946255 10.1111/j.1365-2567.2005.02156.x PMC1782165

[39] Lv L, Lv L, Zhang Y, Kong Q. Luteolin prevents LPS-induced TNF-α expression in cardiac myocytes through inhibiting NF-κB signaling pathway. Inflammation. 2011;34:620–9. 21076936 10.1007/s10753-010-9271-7

[40] Park CM, Song YS. Luteolin and luteolin-7-O-glucoside protect against acute liver injury through regulation of inflammatory mediators and antioxidative enzymes in GalN/LPS-induced hepatitic ICR mice. Nutr Res Pract. 2019;13:473–9. 31814922 10.4162/nrp.2019.13.6.473 PMC6883227

[41] Kou JJ, Shi JZ, He YY, Hao JJ, Zhang HY, Luo DM, et al. Luteolin alleviates cognitive impairment in Alzheimer’s disease mouse model via inhibiting endoplasmic reticulum stress-dependent neuroinflammation. Acta Pharmacol Sin. 2022;43:840–9. 34267346 10.1038/s41401-021-00702-8 PMC8975883

[42] Zhang JX, Xing JG, Wang LL, Jiang HL, Guo SL, Liu R. Luteolin inhibits fibrillary β-Amyloid(1–40)-induced inflammation in a human blood-brain barrier model by suppressing the p38 MAPK-mediated NF-κB signaling pathways. Molecules. 2017;22(3):334. 28245546 10.3390/molecules22030334 PMC6155314

[43] Chen HQ, Jin ZY, Wang XJ, Xu XM, Deng L, Zhao JW. Luteolin protects dopaminergic neurons from inflammation-induced injury through inhibition of microglial activation. Neurosci Lett. 2008;448:175–9. 18952146 10.1016/j.neulet.2008.10.046

[44] Elmazoglu Z, Yar Saglam AS, Sonmez C, Karasu C. Luteolin protects microglia against rotenone-induced toxicity in a hormetic manner through targeting oxidative stress response, genes associated with Parkinson’s disease and inflammatory pathways. Drug Chem Toxicol. 2020;43:96–103. 30207190 10.1080/01480545.2018.1504961

[45] Sternberg Z, Chadha K, Lieberman A, Drake A, Hojnacki D, Weinstock-Guttman B, et al. Immunomodulatory responses of peripheral blood mononuclear cells from multiple sclerosis patients upon in vitro incubation with the flavonoid luteolin: additive effects of IFN-beta. J Neuroinflammation. 2009;6:28. 19825164 10.1186/1742-2094-6-28 PMC2768691

[46] Ahmad S, Jo MH, Ikram M, Khan A, Kim MO. Deciphering the potential neuroprotective effects of luteolin against Aβ(1)-(42)-induced Alzheimer’s disease. Int J Mol Sci. 2021;22(17):9583. 34502488 10.3390/ijms22179583 PMC8430819

[47] Che DN, Cho BO, Kim JS, Shin JY, Kang HJ, Jang SI. Luteolin and apigenin attenuate LPS-induced astrocyte activation and cytokine production by targeting MAPK, STAT3, and NF-κB signaling pathways. Inflammation. 2020;43:1716–28. 32462548 10.1007/s10753-020-01245-6

[48] Fu X, Zhang J, Guo L, Xu Y, Sun L, Wang S, et al. Protective role of luteolin against cognitive dysfunction induced by chronic cerebral hypoperfusion in rats. Pharmacol Biochem Behav. 2014;126:122–30. 25220684 10.1016/j.pbb.2014.09.005

[49] Zhu L, Bi W, Lu D, Zhang C, Shu X, Lu D. Luteolin inhibits SH-SY5Y cell apoptosis through suppression of the nuclear transcription factor-κB, mitogen-activated protein kinase and protein kinase B pathways in lipopolysaccharide-stimulated cocultured BV2 cells. Exp Ther Med. 2014;7:1065–70. 24940388 10.3892/etm.2014.1564 PMC3991549

[50] Aziz N, Kim MY, Cho JY. Anti-inflammatory effects of luteolin: a review of in vitro, in vivo, and in silico studies. J Ethnopharmacol. 2018;225:342–58. 29801717 10.1016/j.jep.2018.05.019

[51] Caporali S, De Stefano A, Calabrese C, Giovannelli A, Pieri M, Savini I, et al. Anti-inflammatory and active biological properties of the plant-derived bioactive compounds luteolin and luteolin 7-glucoside. Nutrients. 2022;14(6):1155. 35334812 10.3390/nu14061155 PMC8949538

[52] Hussain MS, Gupta G, Goyal A, Thapa R, Almalki WH, Kazmi I et al. From nature to therapy: luteolin’s potential as an immune system modulator in inflammatory disorders. J Biochem Mol Toxicol. 2023:e23482. 10.1002/jbt.23482 37530602

[53] Zheng N, Yuan P, Li C, Wu J, Huang J. Luteolin reduces BACE1 expression through NF-κB and through estrogen receptor mediated pathways in HEK293 and SH-SY5Y cells. J Alzheimers Dis. 2015;45:659–71. 25589732 10.3233/JAD-142517

[54] Dragicevic N, Smith A, Lin X, Yuan F, Copes N, Delic V, et al. Green tea epigallocatechin-3-gallate (EGCG) and other flavonoids reduce Alzheimer’s amyloid-induced mitochondrial dysfunction. J Alzheimers Dis. 2011;26:507–21. 21694462 10.3233/JAD-2011-101629

[55] Li J-R, Sun M-J, Ping Q-N, Chen X-J, Qi J-P, Han D-E, Metabolism. Excretion and bioavailability of hydroxysafflor yellow A after oral administration of its lipid-based formulation and aqueous solution in rats. Chin J Nat Med. 2010;8:0233–40.

[56] Liu R, Meng F, Zhang L, Liu A, Qin H, Lan X, et al. Luteolin isolated from the medicinal plant Elsholtzia rugulosa (Labiatae) prevents copper-mediated toxicity in β-amyloid precursor protein Swedish mutation overexpressing SH-SY5Y cells. Molecules. 2011;16:2084–96. 21368720 10.3390/molecules16032084 PMC6259644

[57] Wruck CJ, Claussen M, Fuhrmann G, Römer L, Schulz A, Pufe T et al. Luteolin protects rat PC12 and C6 cells against MPP + induced toxicity via an ERK dependent Keap1-Nrf2-ARE pathway. J Neural Transm Suppl. 2007:57–67. 10.1007/978-3-211-73574-9_9 17982879

[58] Kim S, Chin YW, Cho J. Protection of cultured cortical neurons by luteolin against oxidative damage through inhibition of apoptosis and induction of heme oxygenase-1. Biol Pharm Bull. 2017;40:256–65. 28250268 10.1248/bpb.b16-00579

[59] Lin P, Tian XH, Yi YS, Jiang WS, Zhou YJ, Cheng WJ. Luteolin-induced protection of H_2_O_2_-induced apoptosis in PC12 cells and the associated pathway. Mol Med Rep. 2015;12:7699–704. 26459830 10.3892/mmr.2015.4400

[60] Zhao G, Yao-Yue C, Qin GW, Guo LH. Luteolin from purple perilla mitigates ROS insult particularly in primary neurons. Neurobiol Aging. 2012;33:176–86. 20382451 10.1016/j.neurobiolaging.2010.02.013

[61] Wang HR, Pei SY, Fan DX, Liu YH, Pan XF, Song FX, et al. Luteolin protects pheochromocytoma (PC-12) cells against Aβ (25–35)-induced cell apoptosis through the ER/ERK/MAPK signalling pathway. Evid Based Complement Alternat Med. 2020;2020:2861978. 33335556 10.1155/2020/2861978 PMC7723489

[62] Hu LW, Yen JH, Shen YT, Wu KY, Wu MJ. Luteolin modulates 6-hydroxydopamine-induced transcriptional changes of stress response pathways in PC12 cells. PLoS ONE. 2014;9:e97880. 24846311 10.1371/journal.pone.0097880 PMC4028259

[63] Ali F, Rahul, Jyoti S, Naz F, Ashafaq M, Shahid M, et al. Therapeutic potential of luteolin in transgenic drosophila model of Alzheimer’s disease. Neurosci Lett. 2019;692:90–9. 30420334 10.1016/j.neulet.2018.10.053

[64] He Z, Li X, Wang Z, Cao Y, Han S, Li N, et al. Protective effects of luteolin against amyloid beta-induced oxidative stress and mitochondrial impairments through peroxisome proliferator-activated receptor γ-dependent mechanism in Alzheimer’s disease. Redox Biol. 2023;66:102848. 37597424 10.1016/j.redox.2023.102848 PMC10462892

[65] Qin L, Chen Z, Yang L, Shi H, Wu H, Zhang B, et al. Luteolin-7-O-glucoside protects dopaminergic neurons by activating estrogen-receptor-mediated signaling pathway in MPTP-induced mice. Toxicology. 2019;426:152256. 31381935 10.1016/j.tox.2019.152256

[66] Siddique YH, Jyoti S, Naz F. Protective effect of luteolin on the transgenic drosophila model of Parkinson’s disease. Braz J Pharm Sci. 2018;54.

[67] Guo DJ, Li F, Yu PH, Chan SW. Neuroprotective effects of luteolin against apoptosis induced by 6-hydroxydopamine on rat pheochromocytoma PC12 cells. Pharm Biol. 2013;51:190–6. 23035972 10.3109/13880209.2012.716852

[68] Wu PS, Yen JH, Kou MC, Wu MJ. Luteolin and apigenin attenuate 4-hydroxy-2-nonenal-mediated cell death through modulation of UPR, Nrf2-ARE and MAPK pathways in PC12 cells. PLoS ONE. 2015;10:e0130599. 26087007 10.1371/journal.pone.0130599 PMC4472230

[69] Choi SM, Kim BC, Cho YH, Choi KH, Chang J, Park MS, et al. Effects of flavonoid compounds on β-amyloid-peptide-induced neuronal death in cultured mouse cortical neurons. Chonnam Med J. 2014;50:45–51. 25229015 10.4068/cmj.2014.50.2.45 PMC4161760

[70] Zhou F, Chen S, Xiong J, Li Y, Qu L. Luteolin reduces zinc-induced tau phosphorylation at Ser262/356 in an ROS-dependent manner in SH-SY5Y cells. Biol Trace Elem Res. 2012;149:273–9. 22528780 10.1007/s12011-012-9411-z

[71] Liu F, Xu K, Xu Z, de Las Rivas M, Wang C, Li X, et al. The small molecule luteolin inhibits N-acetyl-α-galactosaminyltransferases and reduces mucin-type O-glycosylation of amyloid precursor protein. J Biol Chem. 2017;292:21304–19. 29061849 10.1074/jbc.M117.814202 PMC5766936

[72] Abbas H, Sayed NSE, Youssef N, Mousa PMEG, Fayez MR. Novel luteolin-loaded chitosan decorated nanoparticles for brain-targeting delivery in a sporadic Alzheimer’s disease mouse model: focus on antioxidant, anti-inflammatory, and amyloidogenic pathways. Pharmaceutics. 2022;14(5):1003. 35631589 10.3390/pharmaceutics14051003 PMC9148113

[73] Rezai-Zadeh K, Douglas Shytle R, Bai Y, Tian J, Hou H, Mori T, et al. Flavonoid-mediated presenilin-1 phosphorylation reduces Alzheimer’s disease beta-amyloid production. J Cell Mol Med. 2009;13:574–88. 18410522 10.1111/j.1582-4934.2008.00344.x PMC2671567

[74] Lin CW, Wu MJ, Liu IY, Su JD, Yen JH. Neurotrophic and cytoprotective action of luteolin in PC12 cells through ERK-dependent induction of Nrf2-driven HO-1 expression. J Agric Food Chem. 2010;58:4477–86. 20302373 10.1021/jf904061x

[75] Lin LF, Chiu SP, Wu MJ, Chen PY, Yen JH. Luteolin induces microRNA-132 expression and modulates neurite outgrowth in PC12 cells. PLoS ONE. 2012;7:e43304. 22916239 10.1371/journal.pone.0043304 PMC3420912

[76] DeTure MA, Dickson DW. The neuropathological diagnosis of Alzheimer’s disease. Mol Neurodegener. 2019;14:32. 31375134 10.1186/s13024-019-0333-5 PMC6679484

[77] Breijyeh Z, Karaman R. Comprehensive review on Alzheimer’s disease: causes and treatment. Molecules. 2020;25(24):5789. 33302541 10.3390/molecules25245789 PMC7764106

[78] Santiago JA, Potashkin JA. The impact of disease comorbidities in Alzheimer’s disease. Front Aging Neurosci. 2021;13:631770. 33643025 10.3389/fnagi.2021.631770 PMC7906983

[79] Yanakiev M, Soper O, Berg DA, Kang E. Modelling Alzheimer’s disease using human brain organoids: current progress and challenges. Expert Rev Mol Med. 2022;25:e3. 36517884 10.1017/erm.2022.40

[80] Shepherd C, McCann H, Halliday GM. Variations in the neuropathology of familial Alzheimer’s disease. Acta Neuropathol. 2009;118:37–52. 19306098 10.1007/s00401-009-0521-4

[81] Chen GF, Xu TH, Yan Y, Zhou YR, Jiang Y, Melcher K, et al. Amyloid beta: structure, biology and structure-based therapeutic development. Acta Pharmacol Sin. 2017;38:1205–35. 28713158 10.1038/aps.2017.28 PMC5589967

[82] Gulisano W, Maugeri D, Baltrons MA, Fà M, Amato A, Palmeri A, et al. Role of amyloid-β and tau proteins in Alzheimer’s disease: confuting the amyloid cascade. J Alzheimers Dis. 2018;64:S611–31. 29865055 10.3233/JAD-179935 PMC8371153

[83] Ballard C, Gauthier S, Corbett A, Brayne C, Aarsland D, Jones E. Alzheimer’s disease. Lancet. 2011;377:1019–31. 21371747 10.1016/S0140-6736(10)61349-9

[84] Small SA, Duff K. Linking abeta and tau in late-onset Alzheimer’s disease: a dual pathway hypothesis. Neuron. 2008;60:534–42. 19038212 10.1016/j.neuron.2008.11.007 PMC2692134

[85] Iqbal K, Grundke-Iqbal I. Alzheimer’s disease, a multifactorial disorder seeking multitherapies. Alzheimers Dement. 2010;6:420–4. 20813343 10.1016/j.jalz.2010.04.006 PMC2946155

[86] Kwon Y. Luteolin as a potential preventive and therapeutic candidate for Alzheimer’s disease. Exp Gerontol. 2017;95:39–43. 28528007 10.1016/j.exger.2017.05.014

[87] Ahmad MH, Fatima M, Mondal AC. Influence of microglia and astrocyte activation in the neuroinflammatory pathogenesis of Alzheimer’s disease: rational insights for the therapeutic approaches. J Clin Neurosci. 2019;59:6–11. 30385170 10.1016/j.jocn.2018.10.034

[88] Facchinetti R, Valenza M, Bronzuoli MR, Menegoni G, Ratano P, Steardo L, et al. Looking for a treatment for the early stage of Alzheimer’s disease: preclinical evidence with co-ultramicronized palmitoylethanolamide and luteolin. Int J Mol Sci. 2020;21(11):3802. 32471239 10.3390/ijms21113802 PMC7312730

[89] Liu R, Lan X, Ying J, Du GH. Protective effects of luteolin against amyloid β25-35-induced toxicity on rat cerebral microvascular endothelial cells. Chin J Nat Med. 2010;8:223–7.

[90] Akasaka-Manya K, Manya H. The role of APP O-glycosylation in Alzheimer’s disease. Biomolecules. 2020;10(11):1569. 33218200 10.3390/biom10111569 PMC7699271

[91] He H, Chen X. Luteolin attenuates cognitive dysfunction induced by chronic cerebral hypoperfusion through the modulation of the PI3K/Akt pathway in rats. J Vet Res. 2021;65:341–9. 34917848 10.2478/jvetres-2021-0037 PMC8643096

[92] Xu B, Li XX, He GR, Hu JJ, Mu X, Tian S, et al. Luteolin promotes long-term potentiation and improves cognitive functions in chronic cerebral hypoperfused rats. Eur J Pharmacol. 2010;627:99–105. 19857483 10.1016/j.ejphar.2009.10.038

[93] Wang H, Wang H, Cheng H, Che Z. Ameliorating effect of luteolin on memory impairment in an Alzheimer’s disease model. Mol Med Rep. 2016;13:4215–20. 27035793 10.3892/mmr.2016.5052 PMC4838167

[94] Liu R, Gao M, Qiang GF, Zhang TT, Lan X, Ying J, et al. The anti-amnesic effects of luteolin against amyloid beta(25–35) peptide-induced toxicity in mice involve the protection of neurovascular unit. Neuroscience. 2009;162:1232–43. 19442706 10.1016/j.neuroscience.2009.05.009

[95] Yu TX, Zhang P, Guan Y, Wang M, Zhen MQ. Protective effects of luteolin against cognitive impairment induced by infusion of Aβ peptide in rats. Int J Clin Exp Pathol. 2015;8:6740–7. 26261557 PMC4525891

[96] Tana NT. Luteolin ameliorates depression-like behaviors by suppressing ER stress in a mouse model of Alzheimer’s disease. Biochem Biophys Res Commun. 2022;588:168–74. 34959189 10.1016/j.bbrc.2021.12.074

[97] Sterniczuk R, Antle MC, Laferla FM, Dyck RH. Characterization of the 3xTg-AD mouse model of Alzheimer’s disease: part 2. Behavioral and cognitive changes. Brain Res. 2010;1348:149–55. 20558146 10.1016/j.brainres.2010.06.011

[98] Park S, Kim DS, Kang S, Kim HJ. The combination of luteolin and l-theanine improved Alzheimer disease-like symptoms by potentiating hippocampal insulin signaling and decreasing neuroinflammation and norepinephrine degradation in amyloid-β-infused rats. Nutr Res. 2018;60:116–31. 30527255 10.1016/j.nutres.2018.09.010

[99] Elsheikh MA, El-Feky YA, Al-Sawahli MM, Ali ME, Fayez AM, Abbas H. sA brain-targeted approach to ameliorate memory disorders in a sporadic Alzheimer’s disease mouse model via intranasal luteolin-loaded nanobilosome. Pharmaceutics. 2022;14(3):576. 35335952 10.3390/pharmaceutics14030576 PMC8950550

[100] Balestrino R, Schapira AHV. Parkinson disease. Eur J Neurol. 2020;27:27–42. 31631455 10.1111/ene.14108

[101] Pringsheim T, Jette N, Frolkis A, Steeves TD. The prevalence of Parkinson’s disease: a systematic review and meta-analysis. Mov Disord. 2014;29:1583–90. 24976103 10.1002/mds.25945

[102] Kim WS, Kågedal K, Halliday GM. Alpha-synuclein biology in lewy body diseases. Alzheimers Res Ther. 2014;6:73. 25580161 10.1186/s13195-014-0073-2 PMC4288216

[103] Ashaari Z, Hadjzadeh MA, Hassanzadeh G, Alizamir T, Yousefi B, Keshavarzi Z et al. The flavone luteolin improves central nervous system disorders by different mechanisms: a review. J Mol Neurosci. 65:491–506. 10.1007/s12031-018-1094-2 30083786

[104] Reudhabibadh R, Binlateh T, Chonpathompikunlert P, Nonpanya N, Prommeenate P, Chanvorachote P, et al. Suppressing Cdk5 activity by luteolin inhibits MPP(+)-induced apoptotic of neuroblastoma through Erk/Drp1 and Fak/Akt/GSK3β pathways. Molecules. 2021;26(5):1307. 33671094 10.3390/molecules26051307 PMC7957557

[105] Nishiguchi H, Omura T, Sato A, Kitahiro Y, Yamamoto K, Kunimasa J, et al. Luteolin protects against 6-hydoroxydopamine-induced cell death via an upregulation of HRD1 and SEL1L. Neurochem Res. 2023;49:117–28. 37632637 10.1007/s11064-023-04019-2 PMC10776467

[106] Dayalu P, Albin RL. Huntington disease: pathogenesis and treatment. Neurol Clin. 2015;33:101–14. 25432725 10.1016/j.ncl.2014.09.003

[107] Ignácio ZM, Quevedo J, Réus GZ. Pathophysiological mechanisms of Huntington’s disease. Pathology. In: Singh S, Joshi N, editors. Prevention and therapeutics of neurodegenerative disease. Singapore: Springer; 2018. pp. 49–60.

[108] Khan H, Ullah H, Tundis R, Belwal T, Devkota H, Daglia M, et al. Dietary flavonoids in the management of Huntington’s Disease: mechanism and clinical perspective. eFood. 2020;1:38–52. 10.2991/efood.k.200203.001

[109] Oliveira AM, Cardoso SM, Ribeiro M, Seixas RS, Silva AM, Rego AC. Protective effects of 3-alkyl luteolin derivatives are mediated by Nrf2 transcriptional activity and decreased oxidative stress in Huntington’s disease mouse striatal cells. Neurochem Int. 2015;91:1–12. 26476055 10.1016/j.neuint.2015.10.004

[110] Hasan Siddique Y, Varshney H, Mantasha I, Shahid M. Effect of luteolin on the transgenic Drosophila model of Huntington’s disease. Comput Toxicol. 2021;17:100148. 10.1016/j.comtox.2020.100148

[111] Chen HI, Hu WS, Hung MY, Ou HC, Huang SH, Hsu PT, et al. Protective effects of luteolin against oxidative stress and mitochondrial dysfunction in endothelial cells. Nutr Metab Cardiovasc Dis. 2020;30:1032–43. 32402583 10.1016/j.numecd.2020.02.014

[112] Bayat P, Farshchi M, Yousefian M, Mahmoudi M, Yazdian-Robati R. Flavonoids, the compounds with anti-inflammatory and immunomodulatory properties, as promising tools in multiple sclerosis (MS) therapy: a systematic review of preclinical evidence. Int Immunopharmacol. 2021;95:107562. 33770729 10.1016/j.intimp.2021.107562

[113] La Rosa G, Lonardo MS, Cacciapuoti N, Muscariello E, Guida B, Faraonio R, et al. Dietary polyphenols, microbiome, and multiple sclerosis: from molecular anti-inflammatory and neuroprotective mechanisms to clinical evidence. Int J Mol Sci. 2023;24(8):7247. 37108412 10.3390/ijms24087247 PMC10138565

[114] Barbierato M, Facci L, Marinelli C, Zusso M, Argentini C, Skaper SD, et al. Co-ultramicronized palmitoylethanolamide/luteolin promotes the maturation of oligodendrocyte precursor cells. Sci Rep. 2015;5:16676. 26578323 10.1038/srep16676 PMC4649338

[115] Facci L, Barbierato M, Fusco M, Giusti P, Zusso M. Co-ultramicronized palmitoylethanolamide/luteolin-induced oligodendrocyte precursor cell differentiation is associated with Tyro3 receptor upregulation. Front Pharmacol. 2021;12:698133. 34276381 10.3389/fphar.2021.698133 PMC8277943

[116] Brotini S. Palmitoylethanolamide/luteolin as adjuvant therapy to improve an unusual case of camptocormia in a patient with Parkinson’s disease: a case report. Innov Clin Neurosci. 2021;18:12–4. 35096476 PMC8794485

[117] Cordaro M, Cuzzocrea S, Crupi R. An update of palmitoylethanolamide and luteolin effects in preclinical and clinical studies of neuroinflammatory events. Antioxid (Basel). 2020;9(3):216. 10.3390/antiox9030216 PMC7139331 32150935

[118] Bang J, Spina S, Miller BL. Frontotemporal dementia. Lancet. 2015;386:1672–82. 26595641 10.1016/S0140-6736(15)00461-4 PMC5970949

[119] Theoharides TC, Asadi S, Panagiotidou S. A case series of a luteolin formulation (NeuroProtek®) in children with autism spectrum disorders. Int J Immunopathol Pharmacol. 2012;25:317–23. 22697063 10.1177/039463201202500201

[120] Taliou A, Zintzaras E, Lykouras L, Francis K. An open-label pilot study of a formulation containing the anti-inflammatory flavonoid luteolin and its effects on behavior in children with autism spectrum disorders. Clin Ther. 2013;35:592–602. 23688534 10.1016/j.clinthera.2013.04.006

[121] Tsilioni I, Taliou A, Francis K, Theoharides TC. Children with autism spectrum disorders, who improved with a luteolin-containing dietary formulation, show reduced serum levels of TNF and IL-6. Transl Psychiatry. 2015;5:e647. 26418275 10.1038/tp.2015.142 PMC5545641

[122] De Luca P, Camaioni A, Marra P, Salzano G, Carriere G, Ricciardi L, et al. Effect of ultra-micronized palmitoylethanolamide and luteolin on olfaction and memory in patients with long COVID: results of a longitudinal study. Cells. 2022;11(16):2552. 36010630 10.3390/cells11162552 PMC9406356

[123] Caltagirone C, Cisari C, Schievano C, Di Paola R, Cordaro M, Bruschetta G, et al. Co-ultramicronized palmitoylethanolamide/luteolin in the treatment of cerebral ischemia: from rodent to man. Transl Stroke Res. 2016;7:54–69. 26706245 10.1007/s12975-015-0440-8 PMC4720704

[124] Park SE, Paudel P, Wagle A, Seong SH, Kim HR, Fauzi FM, et al. Luteolin, a potent human monoamine oxidase-A inhibitor and dopamine D(4) and vasopressin V(1A) receptor antagonist. J Agric Food Chem. 2020;68:10719–29. 32869630 10.1021/acs.jafc.0c04502

[125] Zuiki M, Chiyonobu T, Yoshida M, Maeda H, Yamashita S, Kidowaki S, et al. Luteolin attenuates interleukin-6-mediated astrogliosis in human iPSC-derived neural aggregates: a candidate preventive substance for maternal immune activation-induced abnormalities. Neurosci Lett. 2017;653:296–301. 28595950 10.1016/j.neulet.2017.06.004

[126] Rund BR. Is schizophrenia a neurodegenerative disorder? Nord J Psychiatry. 2009;63:196–201. 19235629 10.1080/08039480902767286

[127] Armstrong R. What causes neurodegenerative disease? Folia Neuropathol. 2020;58:93–112. 32729289 10.5114/fn.2020.96707

[128] Teleanu DM, Niculescu AG, Lungu II, Radu CI, Vladâcenco O, Roza E, et al. An overview of oxidative stress, neuroinflammation, and neurodegenerative diseases. Int J Mol Sci. 2022;23(11):5938. 35682615 10.3390/ijms23115938 PMC9180653

[129] Wu Y, Jiang X, Yang K, Xia Y, Cheng S, Tang Q, et al. Inhibition of α-synuclein contributes to the ameliorative effects of dietary flavonoids luteolin on arsenite-induced apoptotic cell death in the dopaminergic PC12 cells. Toxicol Mech Methods. 2017;27:598–608. 28583009 10.1080/15376516.2017.1339155

[130] Jang S, Dilger RN, Johnson RW. Luteolin inhibits microglia and alters hippocampal-dependent spatial working memory in aged mice. J Nutr. 2010;140:1892–8. 20685893 10.3945/jn.110.123273 PMC2937579

[131] Dirscherl K, Karlstetter M, Ebert S, Kraus D, Hlawatsch J, Walczak Y, et al. Luteolin triggers global changes in the microglial transcriptome leading to a unique anti-inflammatory and neuroprotective phenotype. J Neuroinflammation. 2010;7:3. 20074346 10.1186/1742-2094-7-3 PMC2819254

[132] Paredes-Gonzalez X, Fuentes F, Jeffery S, Saw CL, Shu L, Su ZY, et al. Induction of NRF2-mediated gene expression by dietary phytochemical flavones apigenin and luteolin. Biopharm Drug Dispos. 2015;36:440–51. 25904312 10.1002/bdd.1956

[133] Parrella E, Porrini V, Iorio R, Benarese M, Lanzillotta A, Mota M, et al. PEA and luteolin synergistically reduce mast cell-mediated toxicity and elicit neuroprotection in cell-based models of brain ischemia. Brain Res. 2016;1648:409–17. 27423516 10.1016/j.brainres.2016.07.014

